# A Method Scope Extension for the Simultaneous Analysis of POPs, Current-Use and Banned Pesticides, Rodenticides, and Pharmaceuticals in Liver. Application to Food Safety and Biomonitoring

**DOI:** 10.3390/toxics9100238

**Published:** 2021-09-27

**Authors:** Cristian Rial-Berriel, Andrea Acosta-Dacal, Manuel Zumbado, Luis Alberto Henríquez-Hernández, Ángel Rodríguez-Hernández, Ana Macías-Montes, Luis D. Boada, María del Mar Travieso-Aja, Beatriz Martin Cruz, Octavio P. Luzardo

**Affiliations:** 1 Toxicology Unit, Research Institute of Biomedical and Health Sciences (IUIBS), Universidad de Las Palmas de Gran Canaria, Paseo Blas Cabrera s/n, 35016 Las Palmas de Gran Canaria, Spain; cristian.rial@ulpgc.es (C.R.-B.); andrea.acosta@ulpgc.es (A.A.-D.); manuel.zumbado@ulpgc.es (M.Z.); luis.henriquez@ulpgc.es (L.A.H.-H.); anrodrivet@gmail.com (Á.R.-H.); ana.macias@ulpgc.es (A.M.-M.); luis.boada@ulpgc.es (L.D.B.); beatriz.martin@ulpgc.es (B.M.C.); 2 Study Group on Wild Animal Conservation Medicine (GEMAS), 28040 Madrid, Spain; 3 Spanish Biomedical Research Center in Physiopathology of Obesity and Nutrition (CIBERObn), 28029 Madrid, Spain; 4 Grupo Hospitalario San Roque, C/Dolores de la Rocha, 35001 Las Palmas de Gran Canaria, Spain; marimartravieso@gmail.com

**Keywords:** persistent organic pollutants, agrochemicals, environmental pollution, QuEChERS, LC-MS/MS, GC-MS/MS

## Abstract

The screening of hundreds of substances belonging to multiple chemical classes in liver is required in areas such as food safety or biomonitoring. We adapted a previous QuEChERS-based method in blood to the liver matrix and applied to these fields of study. The validation of the method allowed the inclusion of 351 contaminants, 80% with a LOQ < 2 ng/g. In the analysis of 42 consumer liver samples, we detected trace levels of 29 different contaminants. The most frequent and concentrated was 4,4’-DDE. POPs accounted for 66% of the compounds detected. In no case was the MRL reached for any of the contaminants detected. We also applied the method to 151 livers of wild birds to perform a biomonitoring pilot study in the Canary Islands. We detected 52 contaminants in 15 bird species. These were also mostly POPs, although high frequencies and concentrations of anticoagulant rodenticides (AR) and some other agricultural pesticides also stand out. POPs and AR contamination levels were significantly higher in terrestrial birds, raptors and particularly in nocturnal birds. Pesticide contamination levels were also higher in terrestrial birds, as well as in non-raptors and diurnal birds. The validated method is simple, robust, and sensitive and performs well in a variety of practical scenarios, where it can be carried out relatively quickly and inexpensively.

## 1. Introduction

Animal liver is a common matrix for the search of chemical residues for several reasons. On the one hand, the use of veterinary drugs, which is widely accepted in veterinary practice to treat bacterial infections, parasitism (internal or external), inflammation, and other animal diseases or their symptoms in livestock practice [1,2], may condition the appearance of residues of these chemicals in foods of animal origin, especially in the liver [3,4,5]. This is particularly relevant, since the presence of antimicrobial compounds can induce the spread of drug-resistant pathogenic bacterial strains or produce allergic reactions in humans [3,6]. In addition, pesticide products used in agriculture can leave residues in the raw materials used in the preparation of animal food, and these residues can in turn generate the appearance of residues in food products of animal origin [5], that can pose a serious risk to the health of consumers [6]. Therefore, to protect consumers from these undesirable effects, as a food safety measure, maximum residue limits (MRLs) have been established for many veterinary medicinal products and pesticides on a range of commodities of animal origin, including meat and meat products, liver, fish, honey, milk, and eggs [5,7]. According to the European regulations, the liver must be investigated for the presence of several hundred compounds, including pesticides commonly used in agriculture, pesticides already banned but of great environmental persistence, and also residues of veterinary drugs [7]. Although there are differences between countries, if we take as a common reference what is established in the Codex Alimentarius, there are at least maximum residue limits (MRLs) established for some 218 pesticides and for another 75 veterinary drugs (also including combinations of drugs and animal feed) [4,5].

On the other hand, wildlife lives in an environment that is increasingly contaminated with chemical substances (pesticides, industrial pollutants, wastewater, urban solid waste, etc.) and therefore can serve as first line indicators of the levels of pollutants and their possible health impacts [8,9,10]. Wildlife biomonitoring can provide important information about the bioavailability of contaminants in the environment humans share with these species [11,12,13], which supports the design of appropriate remediation strategies [14]. These data can result in substantial savings of limited remediation resources while maximizing the preservation of important natural areas and supporting effective site remediation [14]. The action of monitoring wildlife exposure to chemical contaminants is usually known as biomonitoring [10,15,16].

Like pesticides and pharmaceuticals, the annual use of anticoagulant rodenticides (ARs) for rodent control is measured in thousands of tons. This extensive use often leads to unintentional exposure of non-target animals, especially birds of prey, to these poisons, so there is a need for these substances to be included in biomonitoring studies. With regard to food safety, it should be noted that ARs are not authorized for use on edible crops within the EU, so the Codex Alimentarius Commission has not set MRLs for any of them, and residues of ARs are not expected to be present in any plant or animal products [17], and therefore they are not usually routinely investigated in food.

Whether it is for residue research in the context of food safety, or in the context of biomonitoring, the liver is an extremely interesting matrix, as it is one of the organs of that concentrates more quantity of chemicals [18]. The range of substances that it is interesting to investigate in one or another circumstance is quite similar, since the substances that are of concern from the point of view of food safety generally also concern from the point of view of the environmental pollution and their effects on wildlife [11,19,20,21]. The availability of multi-residue methods that are capable of accurately and simultaneously identifying and quantifying the concentration of any of these substances subjected to MRLs that may be present in the liver tissue is extremely interesting [5]. Therefore, it is necessary to develop multi-residue methods belonging to multiple chemical classes. In the case of biomonitoring, moreover, the challenge of detecting such a variety of potentially harmful substances in a complex matrix such as the liver is compounded by the fact that the amount of sample available is usually small [16]. 

Although there are numerous published multi-residue/multi-class methods for the determination of chemicals in animal liver, most focus either on the analysis of pesticides [22,23] or on the analysis of certain groups of veterinary drugs [24,25,26]. However, very few of the published methods address the simultaneous analysis of compounds from both classes [27], and are generally limited to a discrete number of compounds. Therefore, to cover the whole spectrum of compounds of interest in any of the fields (food safety and biomonitoring), it is usually necessary to apply several methods in a complementary manner, which consumes time, economic resources, and sample quantity, which may be limited in the case of wildlife. 

The first part of this research consists of a validation of a method scope extension. The original method was developed for whole blood [16,28], and now it has been validated for liver matrix. But more interesting, probably, is the second part of our paper, in which we present and discuss the results of the application of this methodology to the two fields described. On the one hand we analyzed the residues of substances subjected to MRLs in 46 samples of liver intended for human consumption sampled, acquired in markets, supermarkets, and slaughterhouses. On the other hand, we applied the method to the biomonitoring of 151 wildlife specimens from the Canary Islands received in our service from mid-2020 to April 2021. 

## 2. Materials and Methods

### 2.1. Certified Standards and Reagents

Methanol (MeOH, 99.9% purity), acetonitrile (ACN, 99.9% purity), and formic acid (FA, 98.0% purity), all LC-MS grade, were purchased from Honeywell (Charlotte, NC, USA). LC-Grade water (18.2 MΩ/cm) was purified by a MilliQ A10 Gradient system (Millipore, Molsheim, France). Ammonium acetate Optima LC-MS grade was purchased from Fisher (Fisher Scientific UK, Loughborough, UK). QuEChERS Extract Pouch, AOAC Method (6 g de magnesium sulphate and 1.5 g sodium acetate), were purchased in commercial premixes from Agilent Technologies (Palo Alto, CA, USA).

All certified standards (liquid or solid) of all the individual pollutants and deuterated compounds (P-ISs, procedural internal standards) were obtained from A2S—Analytical Standard Solutions (Staint Jean D’Illac, France), Sigma-Aldrich (Augsburg, Germany), CPA Chem (Stara Zagora, Bulgaria), European Pharmacopoeia Reference Standards (Strasbourg, France), Accustandard (New Haven, CT, USA), and Dr. Ehrestorfer (Augsburg, Germany). All standards were from the highest purity available (93.1% to 99.8%). Individual 1 mg/mL stock solutions of each pollutant were prepared either dissolving or diluting certified standards in ACN, MeOH, water, or acetone (according to the solubility of substances), and stored no more than a year at −32 °C. The standard solutions were sorted, grouping by pesticide, pharmaceuticals, COPs, or procedural internal standards (pIS) to get four intermediate solutions at 1 μg/mL/each. Matrix-matched calibration and quality control points were fortified independently, to get 11 points between 0.4 to 40 ng/mL, with 4 quality controls at 1, 4, 10, and 20 ng/mL.

### 2.2. Liver for Method Validation 

For the development, optimization, and validation of the analytical technique, we employed liver samples obtained from chickens of an organic farm. All the chickens were born in this facility, were healthy and had never been exposed to chemicals (no farms or agricultural facilities in the nearby, and no pharmacological treatments, according to the standards of the production mode), to avoid drug interference. The livers were obtained directly from the slaughterhouse, when these animals were slaughtered for consumption, and placed in 50 mL propylene tubes. Upon arrival at the laboratory, these samples were immediately stored at −24 °C until use.

### 2.3. Sample Preparation and Extraction

The QuEChERS method [29] is a matrix dispersion extraction method, which was initially developed for the analysis of pesticides in fruits and vegetables, but has proven to be versatile, allowing the analysis of many other compounds in complex matrices such as blood, milk, meat, eggs, and even soil [30,31]. We applied it to liver samples, for which it is first necessary to homogenize the liver before applying the QuEChERS extraction. For this purpose, one gram of liver sample was weighed into a tube suitable for homogenization with a Precellys Evolution homogenizer (Bertin Technologies, Rockville, Washington D.C., USA), operated at 6500 rpm, 2 × 30 s. After that, when needed, the fortification was performed, either for validation experiments, for calibration curves, or for the preparation of the quality controls (QC). Then, the homogenate was diluted with 4 mL ultrapure water, and one milliliter of the diluted homogenate was placed in a 5 mL Eppendorf tube to be processed. Ten μL of pIS mix (acenaphthene-d10, atrazine-d5, carbendazim-d3, chlorpyrifos-d10, chrysene-d12, cyromazine-d4, diazinon-d10, linuron-d3, PCB 200, phenanthrene-d10, and pirimicarb-d6) was added to all the tubes (either fortified or not) to reach a final concentration of 10 ng/mL. Next, anhydrous magnesium sulfate (480 mg) and sodium acetate (120 mg) were added to each sample tube, followed by 30 s of vortexing and 1 min of vertical manual shaking. Finally, the Eppendorf tubes were centrifuged for 5 min, at 4500 rpm and 2 °C. The supernatant was then filtered through a 0.2 μm Chromafil PET-20/15 syringe filter (polyester, certified for HPLC, Macherey-Nagel, Düren, Germany) into an amber vial directly, for sequential LC and GC-MS/MS analysis. 

### 2.4. Instrumental Analysis

We found that two complementary analyses are required to detect and quantify the 351 compounds that finally could be included in this method. Thus, an analysis by gas chromatography coupled to triple quadrupole mass spectrometry (GC-MS/MS) is needed for the analysis of the most volatile compounds (mainly persistent organic pollutants and some less polar pesticides) and an analysis by liquid chromatography coupled to triple quadrupole mass spectrometry (LC-MS/MS) for the pharmaceuticals, the rodenticides, and the most polar pesticides.

#### 2.4.1. GC-MS/MS

Gas chromatography was employed for the separation of 126 compounds using an Agilent 7890B gas chromatograph (Agilent Technologies, Palo Alto, CA, USA). Two Agilent J&W HP-5MS (5% cross-linked phenyl-methyl-polysiloxane, Agilent Technologies) ultra-inert fused silica capillary columns, with a total length of 30 m (15 + 15), a film thickness of 0.25 μm and 0.25 mm in diameter, were employed for the separations. The columns were joined by means of a purged joint to allow the application of the back-flushing technique that reduces the background noise and extends the column lifetime. An ultra-inert glass wool inlet liner at 250 °C was used at the injection port, and the injection (1.5 µL) was performed in splitless pulsed mode. The gases used were supplied by Linde (Dublin, Ireland), the carrier gas being helium 5.0 (99.999% purity) at a constant flow 1.5 mL/min, and the collision gas being nitrogen 6.0 (99.9999% purity). The initial oven temperature of 80 °C was maintained for 1.8 min, then increased at a rate of 40 °C/min to 170 °C, then increased at a rate of 10 °C/min to 310 °C, and finally maintained for 3 min at 310 °C. The post-run backflush to clean the column was set at 315 °C for 5 min at −5.8 mL/min for the first column, and the final run time at 21.05 min. For the identification and quantification of the compounds, an Agilent 7010 mass spectrometer (Agilent Technologies, Palo Alto, CA, USA) was used. This equipment was operated in the multiple reaction monitoring mode (MRM), with 24-time segments, cycle time between 300 and 600 ms and a dwell time between 15 and 40 ms. The electron impact (EI) and transfer line ionization source temperatures were set at 280 °C, with a solvent delay of 3.7 min.

#### 2.4.2. LC-MS/MS

Liquid chromatography was employed to separate 225 substances using an Agilent 1290 Infinity II UHPLC (Agilent Technologies, Palo Alto, CA, USA). The column was an InfinityLab Poroshell 120 (2.1 mm × 100 mm, 2.7 µm), coupled to an inline filter and an UHPLC guard column with the same characteristics as the analytical column, to protect the column. The gradient of mobile phase A was: 95%—0.5 min; 80%—1 min; 60%—2.5 min; 15%—8 min; 0%—10 to 14 min; 95%—14.01 min. Mobile phase A contained 0.1% FA and 2 mM ammonium acetate in ultrapure water; mobile phase B consisted of 2 mM ammonium acetate in MeOH. 8 μL were injected at a flow rate set at 0.4 mL/min and an oven column temperature of 50 °C. For identification and quantification, an Agilent 6460 mass spectrometer (Agilent Technologies, Palo Alto, CA, USA) was employed. It was operated in the dynamic multiple reaction monitoring mode (dMRM), in both positive and negative polarities, with a cycle time 800 ms, a dwell time of 8 to 60 ms, and a total run time of 18 min. The Agilent Jet Stream Electrospray Ionization Source (AJS-ESI) was operated under the following conditions: gas temperature 190 °C; nebulizer gas flow and pressure were 11 L/min and 26 psi, respectively; the temperature of the sheath gas and the flow were 330 °C and 12 L/min, respectively; and the positive and negative capillary voltages were 3900 V and 2600 V. The drying and desolvation gas was nitrogen provided by the Zefiro 40 nitrogen generator (F-DGSi, Evry, France). Nitrogen 6.0 (99.9999% purity, Linde, Dublin, Ireland) was used as the collision gas.

### 2.5. Validation Procedures

Although this is an extension of the analytical scope of a previous method [16]—in this case a change of matrix—it is necessary to undertake a validation process to verify the capacity of the assay to obtain satisfactory results for the analytes in the new matrix. In this research the validation process included the evaluation of linearity, accuracy, precision, calculation of the limit of quantification (LOQ), uncertainty, and the study of the carryover and matrix effect. For most compounds included in this method, there is no specific guide for method validation. For veterinary drugs and considering liver as a food product of animal origin, the requirements for the methods and validation are presented in the UE’s Regulation 808/2021 [32]. Therefore, we decided to follow this regulation, and also the guide of Standard Practices for Method Validation in Forensic Toxicology (SWGTOX) [33], and the EU’s Directorate-General for Health and Food Safety analytical method validation guide (SANTE) [34].

The linearity of the response was studied by injecting blank liver extract samples spiked with all analytes at 11 levels (range 0.4–40 ng/g) and processed with the method described in Section 2.3 of this section, in quintuplicate. To determine accuracy and precision, % recovery (range 70–120% being acceptable, as specified in the guideline used) and % relative standard deviation (%RSD, values ≤ 20% being acceptable) were calculated, respectively. Recovery and RSD experiments were performed with blank liver samples fortified at least in five quintuplicate concentrations within the working range. For the calculation of the LOQ, matrix-matched calibration curves were prepared in quintuplicate (below 20 ng/g). From these, the lowest concentration level of each analyte that met the criteria for identification, accuracy and precision was considered as the LOQ. For confirmation of compound identity and selectivity, 2 MRM transitions were used, one for quantification (Q) and one for confirmation (q). A maximum deviation of ±30% was tolerated for the ion ratio [35]. Similarly, a maximum deviation of ±0.1 min was established for the retention time.

### 2.6. Samples for the Applicability of the Method

The main objective of this research is to demonstrate the applicability of the validated method in the two fields of application mentioned above: (a) verification of compliance with maximum residue limits in livers intended for human consumption; and (b) biomonitoring of contaminants in wildlife. For this purpose, a set of samples was collected for each of the two independent studies. The samples are described in the following subsections.

#### 2.6.1. Sampling for the Food Safety Study

To verify the applicability of this method for the control of residues subject to MRLs in livers intended for human consumption, 46 liver samples from butcheries, supermarkets, and the general slaughterhouse of Gran Canaria were acquired: 34 samples of beef liver and 12 samples of chicken liver. All the samples, as they were acquired, were transferred to the laboratory and frozen at −20 °C until they were processed.

#### 2.6.2. Sampling for the Biomonitoring Study

The validated method was applied to real samples of wildlife specimens that were received in our laboratory for forensic analyses in the period between September 2020 and May 2021. Thus, we studied a series of 151 fresh liver samples belonging to 15 different species of birds. All the specimens were sent by environmental agents or by the Tafira Fauna Rehabilitation Centre, within the framework of the Strategy for the Prevention and Control of Poisoning in the Canary Islands [36]. All the birds included in this part of the study died from different classes of trauma, and there was no suspicion that they died of poisoning. The species included in this study were: *Accipiter nisus* (*n* = 5); *Ardea cinerea* (*n* = 12); *Asio otus canariensis* (*n* = 34); *Burrhinus oedicnemus* (*n* = 10); *Buteo buteo insularum* (*n* = 12); *Calonectris diomedea* (*n* = 8); *Ciconia ciconia* (*n* = 2); *Corvus corax canariensis* (*n* = 16); *Egretta garzetta* (*n* = 4); *Falco eleanorae* (*n* = 2); *Falco pelegrinoides* (*n* = 6); *Falco tinnunculus canariensis* (*n* = 14); *Larus michaellis* (*n* = 14); *Turdus merula* (*n* = 4); and *Tyto alba* (*n* = 8). The livers, received or extracted at in situ necropsy, were kept at −24ºC until the moment of their processing for analysis. No animals were sacrificed for the purposes of this work.

### 2.7. Statistical Analyses

All statistical analyses were performed with GraphPad Prism v9.2 software (GraphPad Software, CA, USA). The distribution of the variables included in this study was evaluated using the Kolmogorov–Smirnov test. The concentration of most of the contaminants detected did not follow a normal distribution, so the results are expressed in terms of median and range. For this same reason nonparametric tests to check for statistical differences between groups were employed, as these evaluate the median rather than the mean, which is appropriate given the relatively high number of undetected values in some groups. Homogeneity of variance (homoscedasticity) was previously tested using Levene’s test. The Kruskal–Wallis and Mann–Whitney U tests were used as nonparametric tests for overall and pairwise comparisons, respectively. However, as an additional check, pairwise comparisons were also performed using Student’s t-test after logarithmic transformation of the data. A P-value of less than 0.05 (two-tailed) was considered statistically significant. The prevalence of exposure to each contaminant for each species was calculated as the percentage of animals with that residue detected in the liver over the total number of individuals of that species studied. For the study of determinants in the series used for biomonitoring, the response variables considered for comparisons were the amount in the liver of (a) the sum of non-persistent pesticides; (b) the sum of persistent organic pollutants; and (c) the sum of rodenticides.

## 3. Results and Discussion

### 3.1. Method Scope Extension Optimization

In our previous research we optimized and validated a multi-residue method for the analysis of 360 substances (pharmaceuticals, pesticides, rodenticides, and POPs) in blood for biomonitoring purposes [16,28]. Therefore, this is not an ex-novo methodological development, but an extension of the scope of our previously published method to include a new matrix, the liver. However, for a better method performance in this more complex matrix, we considered optimizing the previously established chromatographic conditions, including recalculation of RTs, as well as optimization of MRM transitions to allow for higher sensitivity, as well as adjusting qualifiers and qualifier ratios, and identifying possible interferences with matrix components. The compounds are shown in alphabetical order in Appendix A along with their retention time, transitions, and their collision energies. As we did with the original method in blood, we decided to directly inject the extracts obtained in acetonitrile for LC-MS/MS and GC-MS/MS analyses, without using evaporation and solvent change, to avoid the loss of the more volatile compounds. Several authors, including our group [30,37,38], have shown that ACN, although not the most commonly used solvent in GC-MS/MS, is an appropriate solvent for this type of analysis. 

The final number of validated compounds in this scope extension counts 351 chemicals and metabolites compared to 360 in the previous work. With respect to the original method, there are 18 compounds that met the validation criteria in whole blood, which do not meet the validation criteria when the method is applied to liver samples: acetaminophen, chlorfenapyr, corticosterone 21 acetate, phenbutatin oxide, iprodione, isocarbophos, leptophos, malaoxon, malathion, marbofloxacin, methomyl oxime, N,N,-dimethyl-N-tolylsulfamide, paraoxon ethyl, parathion ethyl, penicillin G, phosmet oxon, piperacillin, and trichlorfon. On the other hand, the opposite occurred with 9 compounds. Dichlorvos, doramectin, metalaxyl, methiocarb-sulfoxide, moxidectin, oxime, pthalimide, pyrimicarb-desmethyl and spirotetramat met the validation criteria in the presence of liver matrix and could therefore be included in the method in liver, whereas in blood they did not and had to be left out. 

### 3.2. Validation Parameters

For confirmation of compound identity and selectivity, 2 MRM transitions were used, one for quantification (Q) and one for confirmation (q). A maximum deviation of ±30% was tolerated for the ion ratio. Similarly, a maximum deviation of ±0.1 min was established for the retention time.

We first studied the linearity of the response by injecting blank liver extract samples spiked with all analytes at 11 levels (range 0.4–40 ng/g) and processed in quintuplicate with the method described in Section 2.3. The linearity study on the response (R^2^), indicated that this was higher than 0.98 for all analytes in the range studied. 

To determine accuracy and precision, % recovery and % relative standard deviation (RSD) was calculated. A recovery within the range 70–120% and RSD values ≤ 20% was considered acceptable, as specified in the guidelines employed [33,34]. Recovery and RSD experiments were performed with blank liver samples fortified at least in four quintuplicate concentrations within the working range. The results of the recovery experiments are presented in Appendix B. Regarding accuracy and precision, most compounds meet the validation criteria for concentrations between their LOQ and the highest level studied (40 ng g^−1^). There were some exceptions where recoveries were outside the above range, especially at the lower concentrations. However, these cases are covered, both in the SANTE guideline and in the SWGTOX working document [33,34], which also accepts as a good validation criterion obtaining recoveries between 60% and 140% at some of the concentrations tested, provided that the RSD is less than 15%. Likewise, in some cases, the recoveries were within the established limits with an RSD slightly higher than 15%, a scenario that is also contemplated in the methodological guidelines, provided that the result is reproducible. As a rule, this second exception applies for concentrations equal to or lower than 4 ng g^−1^. As SANTE analytical guide recommends, the expanded measurement uncertainty (U’) was calculated, from precision and bias, and all analytes presented U’ < 50%, that complies with the requirement. 

For the calculation of the LOQ, matrix-matched calibration curves were prepared in quintuplicate (0.2–20 ng g^−1^). From these, the lowest concentration level of each analyte that met the criteria for accuracy and precision was considered as the LOQ. As in the original method, the LOQ for the analytes included in this scope extension was calculated from five replicates of fortified blank matrix, within the working range. The lowest non-zero calibrator approximation was used to calculate LOQs. This means that the lowest point on the calibration curve that met the identity, bias, and precision criteria was established as the LOQ for a given compound. The LOQs for the 351 liver analytes are shown in Appendix B. The LOQ was set at 0.4 ng g^−1^ for 61 compounds, at 0.8 ng g^−1^ for 82 compounds, at 1.2 ng g^−1^ for 40 compounds, at 1.6 ng g^−1^ for 37 compounds, at 2 ng g^−1^ for 50 compounds, at 4 ng g^−1^ for 46 compounds, at 8.0 ng g^−1^ for 24 compounds, at 12 ng g^−1^ for 5 compounds, at 16 ng g^−1^ for 4 compounds, and at 20 ng g^−1^ for 2 compounds. That is, 76.9% of the compounds included in this method can be reliably and accurately quantified at concentrations below 2 ng g^−1^, making it suitable not only for food safety or poisoning diagnostic studies, but also for biomonitoring studies.

In the original method from which we started it was observed that there was a strong blood matrix effect on about 40% of the analytes. Presumably, a similar situation would occur with the liver matrix. Nevertheless, we decided to include the study of the matrix effect within the validation strategy of this analytical scope extension to prove it, as recommended in the reference guides. All validation assays involve the addition of known concentrations of analytes to the matrix. For the matrix effect study, we worked with the addition of three known concentrations of all analytes (2 ng g^−1^, 10 ng g^−1^, and 20 ng g^−1^) on blank liver extract, and the quantification was done against calibration curves prepared in solvent (without matrix). Experiments were performed in quintuplicate for each concentration. One difficulty was that, given the enormous number of substances included in the method, the liver was not completely free of 100% of the chemicals, in particular POPs. Therefore, in these cases, the response of the white matrix sample was subtracted from the calibration standards and QC to calculate the response of the externally added analyte. As we expected, matrix effect (ME) was observed for both, compounds analyzed by LC-MS/MS and GC-MS/MS, especially for compounds analyzed by the latter technique. A strong or medium suppression of the signal was demonstrated for 17.66% of the compounds (*n* = 62), and signal enhancement was verified for 36.47% of the compounds (*n* = 128). For the remaining 45.87% (*n* = 161 contaminants, the ME was considered negligible (−20% < M < 20%). Since for most of the compounds, significant ME was indeed observed, and it was concluded that matrix-matched calibration had to be used to compensate for these interferences. All detailed ME data for individual compounds in liver are shown in Appendix C.

Finally, we also assessed if carryover occurred after injecting a blank matrix fortified at 80 ng g^−1^ and processed with this method, before a blank matrix extract. We were not able to find a clear response in that blank matrix, so we concluded that in our working range, we had not any carryover effect in any of the analyzed compounds.

### 3.3. Application to Food Safety

In the study of the 34 beef liver samples, the results indicated the presence of a discrete number and concentration of contaminants, which ranged from 0 to 15 residues per sample, with an average of 3.13 residues. Of the 351 contaminants and metabolites included in the method, only 25 were detected in the total of beef liver samples, and of these 19 belong to the group of persistent or semi-persistent contaminants (4,4’-DDE, 4,4’-DDD, Dieldrin, Hexachlorobenzene, beta-hexachlorocyclohexane, BDE 153, PCB congeners #105, 118,126, 138, 155, 156, 157, 180, 189, naphthalene, phenanthrene, and pyrene). It is noteworthy that none of the concentrations in any of the samples exceeded the MRL, or even the value of half the MRL. In general, the concentrations of the contaminants detected were low, with the highest values being those of 4,4’-DDE, which was detected in 65.2% of the samples and with a median value of 92.2 ng g^−1^. The relatively high levels of DDT derivatives may seem surprising, as this substance was banned in Spain almost 5 decades ago. However, there is abundant literature that has documented that this pesticide was widely used in the Canary archipelago, and how this translates into the levels of this pesticide detected in food for human consumption produced in this region [39,40,41,42].

The next in frequency and concentration were PCB 153 (26.1%; 35.2 ng g^−1^) and PCB 138 (21.8%; 24.5 ng g^−1^). The other contaminants were detected in frequencies and concentrations much lower than these. Among the non-persistent pesticides detected in this series of consumption livers, very low levels of bifenthrin, fenazaquin, fluquinconazole, flutalonil, flutriafol, and imidacloprid were detected.

If the detection of residues in beef liver was low and of little toxicological relevance, it was even more so in chicken liver. In the 12 samples analyzed, we detected only four contaminants out of the 351 included in the method: fenpropidin, fenpropimorph, levamisole, and 4,4’ DDE. The latter was the more relevant, and it was only detected in three of the 12 livers analyzed and at a much lower concentration than that detected in beef liver (mean = 4.3 ng g^−1^).

Although it is not the main objective of this study, we made an estimate of the risk of exposure to these contaminants through liver consumption. The calculations were made according to the standard methodology that has been described previously [43], and in no case were the tolerable daily intake levels for these contaminants exceeded, mainly due to the low consumption of liver by the Spanish population (only 1 g/day for the total offal consumption) [44].

### 3.4. Application to Biomonitoring

Regarding biomonitoring of chemical substances, this method was applied to fresh livers obtained from 151 carcasses of 15 species of wild birds whose causes of death were not related to poisoning (mainly trauma). Table 1 shows the results for each of the species, limited to show only the 52 contaminants that were detected in the series. This represents that 15% of the contaminants included in the method were detected. 

**Figure 1:**
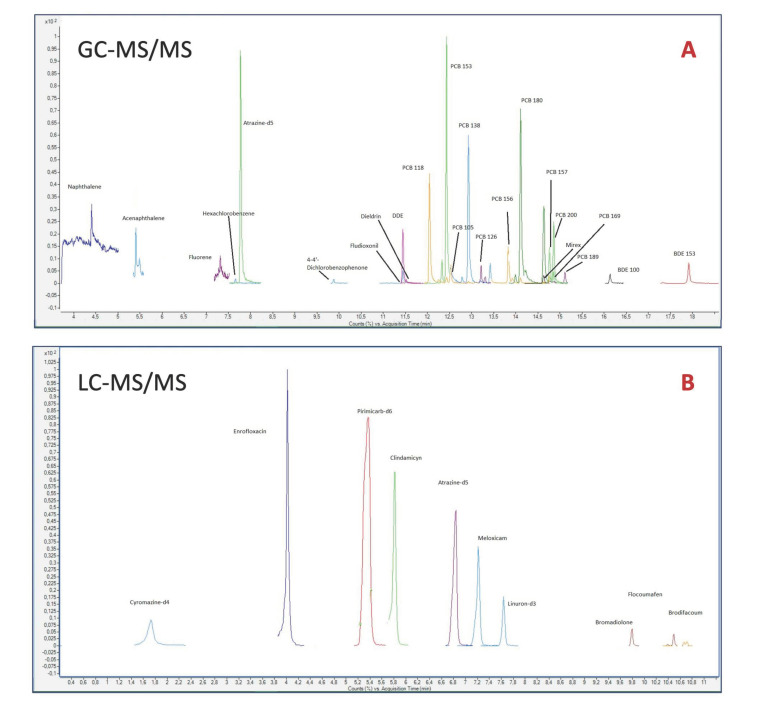
MRM chromatograms of the complementary analyses of a real sample (long-eared owl) by GC-MS/MS (**A**) and by LC–MS/MS (**B**).

The mean value of the number of contaminants per sample was 17. The species with the greatest variety of residues detected was *Asio otus* (*n* = 41), followed by *Falco tinnunculus (n* = 27). In contrast, the species with the lowest number of liver contaminants were *Turdus merula* (*n* = 5) and *Ciconia ciconia (n* = 3). Figure 1 shows the LC-MS/MS and GC-MS/MS chromatograms of one of the birds in the series with the highest number of different contaminants (a long-eared owl). 

**Figure 2:**
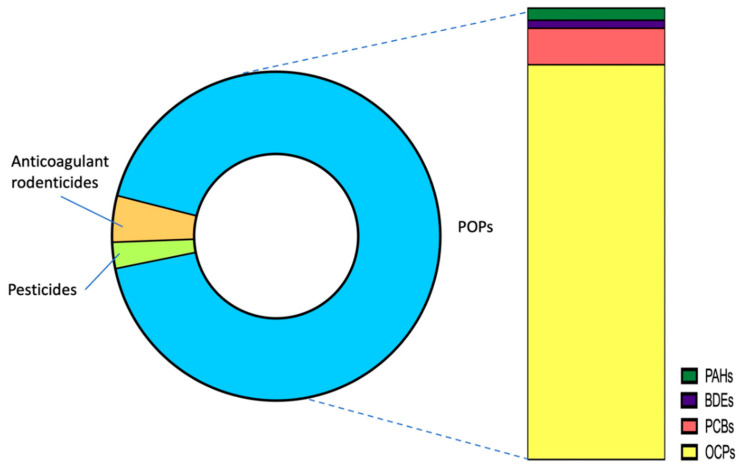
Occurrence of environmental pollutants in the liver of a series of 151 wild birds of the Canary Islands.

The most frequently detected contaminant was 4,4’-DDE, which was detected in 138 birds (91.4%), followed by PCB 153, detected in 116 animals (76.8%), brodifacoum in 109 animals (72.2%), bromadiolone in 87 animals (57.6%), and dieldrin in 59 animals (39.1%). With respect to concentrations, the highest concentrations corresponded to enrofloxacin, clindamycin and meloxicam (Table 1). However, these values cannot be considered within the biomonitoring study, since they correspond to drugs used during the hospitalization of many of these animals. Therefore, high concentrations of these substances have been marked with an asterisk. However, other veterinary pharmaceuticals detected in some specimens, such as tetraconazole, metronidazole, or sulfathiazole, are not part of the treatment administered and should be considered contaminants. In general terms, the highest concentrations of contaminants corresponded to 4,4’-DDE in all species. Overall, in quantitative terms, the group of organochlorine pesticides was the most abundant (Figure 2), and the group of persistent and semi-persistent organic pollutants accounted for more than 92% of the total concentration of pollutants detected in the livers of wild birds sampled in the Canary Islands very recently (September 2020–May 2021). This reflects, once again, that contamination by these compounds, in particular organochlorine insecticides, is still very prevalent in the Canary Islands, as has been reported for wildlife in this region [28,45,46,47]. As indicated in the previous section, there is a large literature body documenting the high levels of contamination by organochlorine pesticides in this region [48,49,50], which also translates into high levels in the biota that inhabit the archipelago. There is a possibility that the high levels detected could also come from the neighboring African continent [51], but in this biomonitoring study this option is ruled out, since all the birds sampled for this pilot study are residents in the archipelago and not migratory birds.

With respect to non-persistent pollutants, several aspects should be highlighted. First, the high prevalence of second-generation anticoagulant rodenticides in wildlife’s liver is noteworthy. It was expected, as it has been described in many parts of the world [52,53,54] and recently in the Canary Islands [45,55,56]. However, the presence of at least one of these compounds in more than 80% of the birds studied is striking, even in non-predatory birds such as the blackbird (*Turdus merula*) or the common curlew (*Burhinus oedicnemus*), which would point to the fact that these compounds penetrate the trophic chain by several routes, probably including invertebrates, as suggested by other authors [57,58]. 

The result for carbofuran is also surprising, given that none of the birds studied had any suspicion of intoxication. However, this potent insecticide, banned in the EU since 2007 [59] was detected in small concentrations in the liver of 10 birds of the series, being higher in the case of canary crows (6/12 positives, median = 94.5 ng/g). In all these crows, the main carbofuran metabolite was also detected. This toxicant has widely affected wildlife worldwide [60,61,62,63] and in the Canary Islands its use has been extensive and also has affected wildlife in the past [64]. From the results of this study, it still is today, and it can be concluded that it even penetrates the trophic chain. With respect to the rest of the non-persistent compounds detected in this series, 2-phenylphenol (PHP) stands out. PHP was detected in eleven birds, including five common curlews (Table 1). PHP is a biocide used as a preservative and surface disinfectant on fibers and other materials in homes, hospitals, and elsewhere, and is recognized as a potential endocrine disruptor [65]. Other authors have also reported that PHP is a highly prevalent contaminant in biota samples, such as river fish of different species, where it is found in up to 100% of samples [66]. 

**Figure 3:**
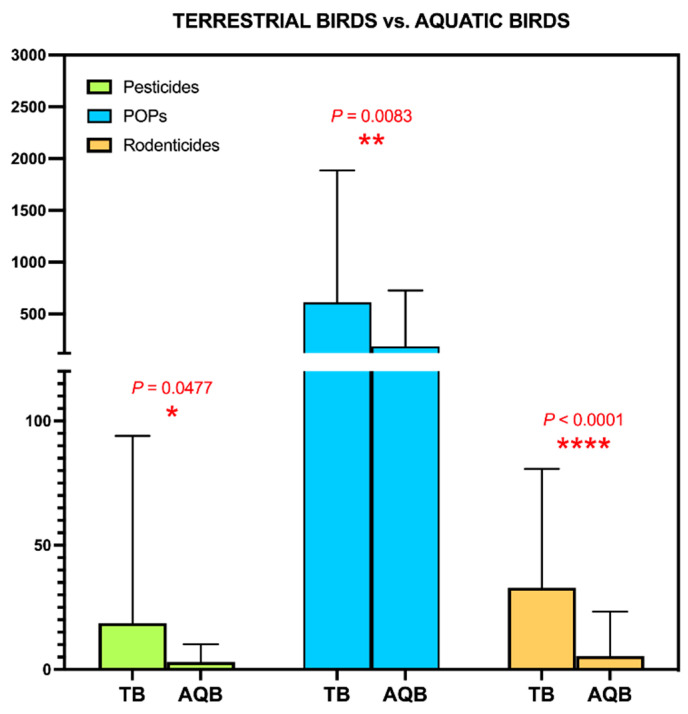
Study of the determinants of environmental contamination detected in the livers of wild birds in the Canary Islands: Habitat type (terrestrial (TB) vs. aquatic birds (AQB)).

Since this was an opportunistic study on carcasses obtained from wildlife recovery centers, we did not have too many quality variables to carry out an in-depth study of the determinants of contamination patterns. Even so, we wanted to explore the influence of the variables inherent to the species studied and found a series of statistically significant differences. Thus, when we compared aquatic versus terrestrial birds, we found that the latter presented significantly higher levels of contamination by the three major chemical groups studied (Figure 3). 

This result was expected with regard to rodenticides, since in a previous study by our group focused on these compounds, we had already discarded the group of waterfowl due to their low incidence in these pollutants [56]. Regarding POPs and non-persistent pesticides, although there is not much literature comparing both types of birds from the same region, the available studies usually indicate results similar to ours, with levels in landbirds usually being higher than in waterbirds [67,68,69]. 

**Figure 4:**
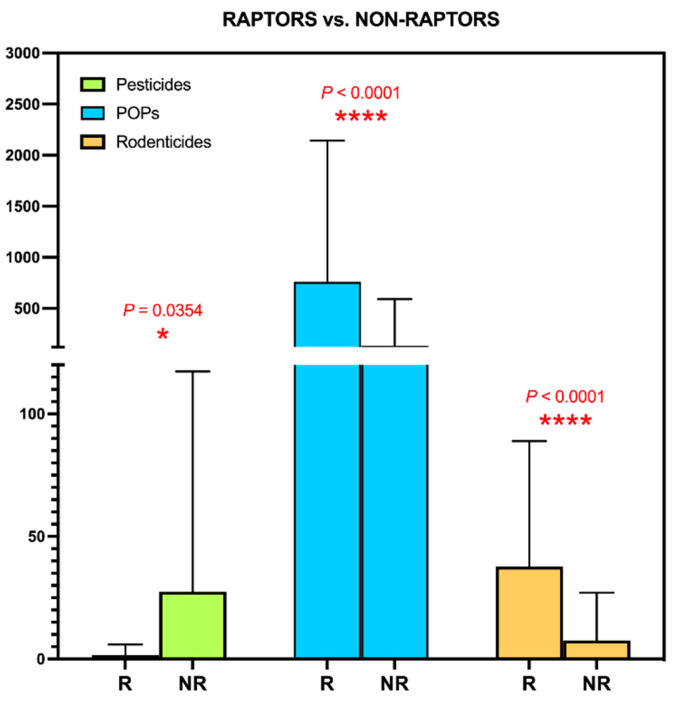
Study of the determinants of environmental contamination detected in the livers of wild birds in the Canary Islands: Diet type (raptors vs. non-raptors).

Another variable that seems to influence the pattern of contamination is the raptor/predator bird status. The raptors in our study presented higher levels of POPs than non-predatory birds (Figure 4), which is logical given that they feed higher in the trophic chain, and has been described in the literature [68,69]. They also presented higher levels of AR, as we expected from having previously observed it in this region [56], and also described by other authors [70]. However, in the case of agricultural pesticides the statistical significance was the opposite, with non-predatory birds presenting the highest levels. There is not much literature to support this finding, but a recent study using the terrestrial pesticide residue exposure (T-REX) model estimated that the highest risk was presented by insectivorous birds, followed by fruit and seed feeders [71]. 

**Figure 5:**
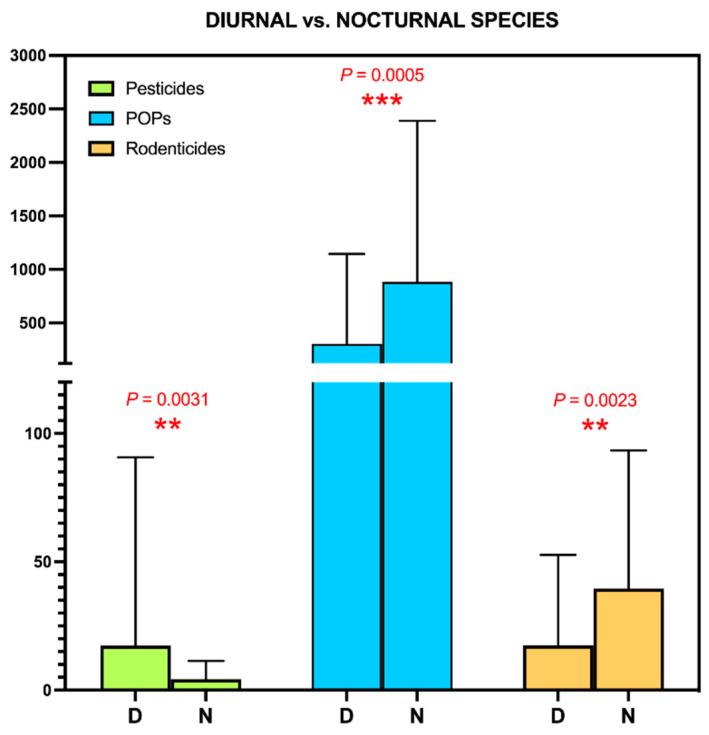
Study of the determinants of environmental contamination detected in the livers of wild birds in the Canary Islands: Habit type (diurnal vs. nocturnal).

Finally, we also studied the influence of the diurnal/nocturnal habits of the birds in the study, and found that diurnal species have higher pesticide levels, but lower POPs and ARs than nocturnal species (Figure 5). We believe that the pesticide result has to do with the previous variable, in the sense that, in our study, all insectivorous species, and those that feed on fruits and seeds are diurnal, while the nocturnal birds in our series are both raptors that feed mainly on large and small rodents. For this same reason, and as we had already verified in previous studies, the nocturnal birds of the Canary Islands have higher levels of POPs [72] and AR [45,56].

## 4. Conclusions

The validated method allows the simultaneous analysis in liver of 351 substances (POPs, pesticides including rodenticides and drugs), using only 1 gram of sample. This is important, since in veterinary forensic medicine, especially with small animals, the amount of sample available is very limited. The proposed analytical method can detect trace amounts of all chemicals in the liver of multiple species. Therefore, it can be successfully applied and used as a routine method in environmental chemistry and forensic toxicology laboratories. The method we have developed can also be used in residue control studies in food intended for human consumption and for the purpose of food safety assessment.

## Figures and Tables

**Table 1:** Median concentrations and frequencies (between parentheses) of organic pollutants detected in wild birds of the Canary Islands. All the results are in ng/g.

Compound	Accipiter Nisus (*n* = 5)	Ardea Cinerea (*n* = 12)	Asio Otus (*n* = 34)	Burrinus Oecdinemus (*n* = 10)	Buteo Buteo (*n* = 12)	Calonectris Diomedea (*n* = 8)	Ciconia Ciconia (*n* = 2)	Corvus Corax (*n* = 16)	Egretta Garzetta (*n* = 4)	Falco Eleonorae (*n* = 2)	Falco Pelegrinoides (*n* = 6)	Falco Tinnunculus (*n* = 14)	Larus Michaelis (*n* = 14)	Turdus Merula (*n* = 4)	Tyto Alba (*n* = 8)
Meloxicam	9.9 (40)	-	47.8 (12)	880.3 * (20)	-	-	-	10.3 (13)	-	-	-	96.1 (29)	-	-	-
Tetraconazole	-	-	0.6 (6)	-	-	-	-	-	-	-	-	-	-	-	-
Clindamycin	1.3 (40)	-	1423 * (18)	1.4 (20)	28.4 (33)	-	-	-	-	-	2.3 (33)	-	-	-	-
Enrofloxacin	5300 * (80)	-	4739 * (29)	4638 * (40)	5453 * (67)	-	-	1970 * (13)	-	20.4 (100)	5144 * (50)	5531 * (57)	10234 * (7)	-	14508 * (50)
Metronidazole	-	-	-	50.8 (10)	-	-	-	-	-	-	-	603.3 (14)	-	-	-
Sulfatiazole	-	-	15.5 (6)	-	-	-	-	-	-	-	-	-	-	-	-
2-Phenylphenol	-	-	16.3 (18)	22.7 (20)	2.4 (17)	-	-	-	-	-	-	-	-	-	-
Boscalid (formerly nicobifen)	-	-	-	-	-	-	-	-	-	-	-	-	-	-	0.2 (25)
Fludioxonil	-	-	0.4 (6)	-	-	-	-	-	-	-	-	-	-	-	-
Fluquinconazole	-	-	-	-	-	-	-	-	-	-	-	-	-	-	-
Flutriafol	-	-	-	-	4.4 (17)	-	-	-	-	-	-	-	-	-	-
Carbofuran	-	-	10.3 (3)	16.4 (10)	7.2 (26)	-	-	94.5 (38)	-	-	-	-	-	-	-
Carbofuran-3-hydroxy	-	-	-	-	-	-	-	2.1 (38)	-	-	-	-	-	-	-
Fipronil	-	-	1.4 (12)	-	-	-	-	-	-	-	-	-	-	-	-
Fipronil sulfide	-	-	3.0 (6)	-	-	-	-	-	-	-	-	-	-	-	-
Permethrin	-	-	-	-	-	23.4 (13)	-	-	-	-	-	-	12.3 (7)	-	-
Acenaphthene	2.4 (80)	1.4 (17)	0.8 (18)	-	0.4 (17)	-	-	-	-	-	-	2.4 (14)	-	-	-
Anthracene	1.5 (40)	-	-	-	-	-	-	-	-	-	-	-	-	-	-
Chrysene	-	-	-	-	-	-	-	-	-	-	-	-	-	-	4.8 (25)
Fluoranthene	0.4 (40)	-	-	-	-	-	-	-	-	-	-	-	-	-	1.7 (25)
Fluorene	5.9 (80)	-	2.5 (12)	-	-	-	-	-	-	-	-	5.3 (7)	-	-	-
Naphtalene	1.8 (100)	-	2.2 (6)	5.7 (40)	3.9 (26)	16.6 (13)	-	3.4 (26)	0.7 (25)	-	-	1.8 (14)	-	0.9 (50)	8.0 (25)
Phenanthrene	13.3 (100)	-	7.5 (12)	-	-	0.4 (13)	-	2.7 (13)	-	-	-	7.6 (28)	-	-	851.3 (50)
Pyrene	1.8 (40)	-	0.7 (9)	-	-	-	-	-	-	-	-	20.0 (28)	-	-	-
4,4′-Dichlorobenzophenone (metabolite of dicofol)	-	-	-	-	-	-	-	-	-	-	-	-	-	-	-
BDE-100	-	1.9 (33)	0.2 (6)	-	-	-	-	-	-	-	0.4 (33)	-	0.2 (7)	-	-
BDE-153	-	0.3 (33)	4.9 (41)	-	-	1.1 (13)	-	-	-	0.7 (50)	19.0 (67)	0.6 (29)	-	-	0.8 (25)
BDE-154	-	2.1 (17)	2.6 (6)	-	-	-	-	-	-	-	6.9 (33)	-	-	-	-
BDE-183	-	-	0.9 (12)	-	-	-	-	-	-	-	-	-	-	-	-
BDE-47	-	1.5 (17)	2.4 (6)	-	-	-	-	-	-	-	-	0.6 (7)	0.4 (14)	-	-
BDE-99	-	-	1.5 (29)	-	-	-	-	-	-	-	3.6 (33)	0.4 (14)	0.6 (14)	7.5 (100)	0.4 (13)
Dichlorodiphenyldichloroethane (p,p′ DDD)	1.2 (80)	-	-	-	-	-	-	-	-	-	-	2.2 (21)	-	-	-
Dichlorodiphenyldichloroethylene (p,p′ DDE)	211.1 (100)	21.1 (100)	305.6 (100)	25.9 (60)	5.6 (83.3)	16.6 (100)	-	6.7 (75)	4.7 (100)	68.4 (100)	318.6 (100)	45.3 (100)	4.4 (100)	-	24.1 (100)
Dieldrin	7.8 (80)	3.5 (17)	5.6 (41.2)	3.0 (10)	0.9 (33)	3.1 (13)	-	-	2.1 (100)	5.8 (100)	8.5 (100)	11.9 (100)	2.3 (7)	-	1.2 (75)
Hexachlorobencene	-	1.4 (34)	0.6 (24)	-	-	7.1 (13)	-	-	12.9 (50)	0.6 (50)	-	1.1 (7)	1.1 (7)	-	0.8 (25)
Hexachlorocyclohexane (beta)	-	-	31.0 (12)	-	-	-	-	-	-	-	3.4 (50)		-	-	-
Mirex	-	-	3.9 (12)	3.6 (20)	3.1 (17)	25.0 (13)	-	-	-	-	2.3 (33)		-	-	-
PCB 105	-	1.2 (50)	1.3 (35)	-	-	2.0 (26)	-	-	3.6 (25)	0.6 (50)	0.6 (67)	1.1 (14)	0.4 (7)	-	-
PCB 118	0.5 (40)	5.4 (50)	4.8 (35)	-	0.5 (17)	45.1 (13)	-	-	14.9 (50)	5.0 (100)	2.2 (100)	1.3 (28)	1.4 (7)	-	1.2 (37)
PCB 138	1.4 (80)	7.9 (100)	2.9 (76)	2.2 (30)	1.3 (34)	8.8 (75)	-	3.9 (38)	24.1 (100)	15.3 (100)	7.7 (100)	4.2 (71)	2.7 (72)	-	5.3 (75)
PCB 153	3.4 (80)	15.5 (100)	3.6 (94)	0.5 (80)	3.3 (34)	6.8 (100)	-	1.7 (88)	55.3 (100)	115.3 (100)	16.0 (100)	5.7 (71)	-	-	7.8 (100)
PCB 156	-	2.0 (50)	2.0 (33)	-	-	8.5 (13)	-	-	0.8 (25)	8.8 (50)	0.8 (100)	0.9 (7)	-	-	0.5 (25)
PCB 157	-	0.8 (17)	1.1 (6)	-	-	2.1 (13)	-	-	9.1 (50)	1.1 (50)	-	-	0.4 (7)	-	-
PCB 167	-	1.7 (50)	1.5 (35)	-	-	6.7 (50)	-	-	44.6 (100)	6.7 (50)	1.3 (67)	0.8 (14)	2.2 (72)	-	0.4 (13)
PCB 180	3.9 (80)	24.3 (67)	3.2 (88)	-	2.1 (50)	3.6 (100)	-	2.8 (88)	-	123.6 (100)	19.8 (100)	5.2 (71)	-	-	8.0 (75)
PCB 189	-	-	2.1 (6)	1.0 (80)	-	-	-	-	-	1.8 (50)	-	-	-	-	-
PCB 28	-	-	5.8 (3)	-	-	-	-	-	-	-	-	-	-	-	-
Brodifacoum	1.7 (100)	0.4 (100)	32.9 (100)	2.3 (80)	0.9 (100)	-	-	27.4 (75)	-	-	20.4 (100)	8.8 (50)	1.4 (21)	-	20.31 (100)
Bromadiolone	-	-	1.3 (100)	2.1 (100)	8.5 (100)	-	-	2.25 (38)	-	1.1 (50)	4.6 (100)	2.5 (75)	-	0.34 (25)	2.2 (75)
Difenacoum	-	0.8 (17)	0.6 (24)	-	1.5 (50)	-	-	0.9 (13)	-	-	0.9 (33)	1.2 (57)	-	-	3.6 (25)
Difethialone	-	-	18.9 (18)	-	-	-	-	-	-	-	-	1.9 (29)	-	-	-
Flocoumafen	-	-	0.7 (24)	-	4.1 (17)	-	-	-	-	-	-	2.2 (7)	-	-	-

* These values cannot be considered as biomonitoring, since these pharmaceuticals were employed during the treatment of the animals at the Wildlife Recovery Centers.

## Data Availability

On request to the authors.

## Appendix A

**Table A1:** Chromatographic and mass spectrometric conditions of the compounds analyzed in liver.

No.	Compound	Technique	Retention Time (min)	Polarity	Quantification		Confirmation		Fragmentor Voltage (V)
					MRM (m/z)	Collision energy (eV)	MRM transition (m/z)	Collision energy (eV)	
1	2-Phenylphenol	GC	6.28	positive	169.0 → 115.0	30	169.0 → 141.0	15	70
2	4,4’-Dichlorobenzophenone (metabolite of dicofol)	GC	9.99	positive	250.0 → 139.0	15	250.0 → 215.0	5	70
3	Abamectine	LC	10.99	positive	890.5 → 567.1	10	895.5 → 751.4	45	160
4	Acenaphthene	GC	6.15	positive	153.0 → 152.0	25	153.0 → 151.0	35	70
5	Acenaphtylene	GC	5.94	positive	152.0 → 151.0	25	152.0 → 126.0	30	70
6	Acephate	LC	1.64	positive	184.0 → 143.0	15	143.0 → 95.0	15	70
7	Acetamiprid	LC	4.43	positive	223.1 → 126.0	27	223.1 → 90.0	45	140
8	Acrinathrin	LC	10.70	positive	559.0 → 208.0	10	559.0 → 181.0	30	70
9	Albendazole	LC	7.14	positive	266.1 → 234.1	16	266.1 → 191.0	32	155
10	Aldicarb	LC	5.11	positive	208.0 → 116.0	10	116.0 → 89.1	4	100
11	Aldicarb-sulfone	LC	3.21	positive	240.1 → 76.0	16	223.1 → 86.1	13	75
12	Aldicarb-sulfoxide	LC	2.75	positive	207.1 → 131.9	10	207.1 → 89.1	10	86
13	Aldrin	GC	9.90	positive	255.0 → 220.0	25	263.0 → 228.0	10	70
14	Anthracene	GC	8.40	positive	178.0 → 176.0	35	178.0 → 152.0	30	70
15	Atrazine	LC	6.73	positive	216.0 → 173.9	15	216.0 → 103.8	30	130
16	Azinphos-methyl	LC	7.27	positive	318.0 → 132.1	8	340.0 → 160.0	10	60
17	Azoxystrobin	LC	7.59	positive	404.1 → 372.1	8	404.1 → 344.1	24	110
18	BDE-28	GC	12.22	positive	406.0 → 246.0	20	406.0 → 167.0	25	70
19	BDE-47	GC	14.31	positive	326.0 → 138.0	45	484.0 →324.0	25	70
20	BDE-85	GC	17.08	positive	564.0 → 404.0	25	566.0 → 406.0	25	70
21	BDE-99	GC	16.27	positive	566.0 → 406.0	25	564.0 → 404.0	30	70
22	BDE-100	GC	15.85	positive	566.0 → 406.0	25	564.0 → 404.0	25	70
23	BDE-153	GC	18.04	positive	644.0 → 484.0	25	486.0 → 377.0	30	70
24	BDE-154	GC	17.47	positive	644.0 → 484.0	25	486.0 → 377.0	30	70
25	BDE-183	GC	20.12	positive	561.6 → 454.7	40	563.6 → 454.7	40	70
26	Benalaxyl	LC	8.96	positive	326.2 → 148.0	20	326.2 → 208.0	12	90
27	Bendiocarb	LC	5.88	positive	224.1 → 166.9	8	224.2 → 108.9	15	120
28	Bendiocarb metabolite (2,2-dimethylbenzo-1. 3-dioxol-4-ol)	GC	4.84	positive	166.0 → 151.0	10	166.0 → 126.0	20	70
29	Benfuracarb	LC	9.73	positive	411.2 → 190.0	13	411.2 → 252.0	15	110
30	Benzo[a]anthracene	GC	13.95	positive	228.0 → 226.0	40	228.0 → 202.0	35	70
31	Benzo[a]pyrene	GC	16.89	positive	252.0 → 250.0	45	252.0 → 248.0	60	70
32	Benzo[b]fluoranthene	GC	16.30	positive	252.0 → 248.0	60	252.0 → 226.0	35	70
33	Benzo[ghi]perylene	GC	19.61	positive	276.0 → 274.0	50	276.0 → 272.0	60	70
34	Benzo[k]fluoranthene	GC	16.29	positive	252.0 → 250.0	45	252.0 → 224.0	40	70
35	Bifenthrin	GC	11.25	positive	440.0 → 181.0	5	440.0 → 165.0	60	94
36	Bitertanol	LC	9.23	positive	338.2 → 70.0	4	338.2 → 269.2	5	100
37	Boscalid (formerly nicobifen)	GC	7.84	positive	3434.0 → 272.0	30	343.0 → 140.0	45	100
38	Brodifacoum	LC	10.78	negative	521.3 → 79.0	50	523.3 → 135.0	45	220
39	Bromadiolone	LC	9.75	negative	525.3 → 250.0	40	527.3 → 250.0	40	200
40	Bromopropylate	GC	13.87	positive	341.0 → 183.0	15	341.0 → 157.0	45	70
41	Bromuconazole (two isomers)	GC	13.81/14.24	positive	295.0 → 173.0	10	295.0 → 175.0	10	70
42	Bupirimate	LC	11.78	positive	273.0 → 108.0	15	273.0 → 193.0	5	70
43	Buprofezin	LC	9.83	positive	306.1 → 201.0	12	306.1 → 116.0	12	140
44	Cadusafos (ebufos)	LC	9.39	positive	271.1 → 159.0	16	271.1 → 131.0	22	100
45	Carbaryl	LC	6.21	positive	202.1 → 145.1	4	202.1 → 127.1	28	95
46	Carbendazim (azole)	LC	2.90	positive	192.1 → 160.1	4	202.1 → 127.1	28	90
47	Carbofuran	LC	5.91	positive	222.1 → 123.1	20	222.1 → 165.1	30	80
48	Carbofuran-3-hydroxy	LC	4.27	positive	238.1 → 163.1	10	238.1 → 181.1	10	110
49	Carbosulfan	LC	11.03	positive	381.2 → 160.2	12	381.2 → 76.1	36	120
50	Cefuroxima axetil (two isomers)	LC	5.13	positive	533.0 → 447.0	15	533.0 → 386.0	20	160
51	Chloramphenicol	LC	4.63	negative	321.0 → 152.1	4	323.0 → 152.1	4	113
52	Chlorantraniliprole	LC	7.32	positive	483.9 → 452.9	16	483.9 → 285.9	8	105
53	Chlorfenvinphos	LC	9.09	positive	361.1 → 98.9	34	358.9 → 155.1	8	105
54	Chlorobenzilate	GC	12.14	positive	251.0 → 111.0	40	251.0 → 139.0	15	70
55	Chlorophacinone	LC	8.88	negative	373.2 → 201.0	20	375.2 → 203.0	20	160
56	Chlorpropham	GC	7.13	positive	213.0 → 127.0	15	153.0 → 90.0	25	70
57	Chlorpyrifos	GC	9.93	positive	314.0 → 258.0	15	314.0 → 286.0	5	70
58	Chlorpyrifos methyl	GC	9.12	positive	286.0 → 93.0	25	286.0 → 271.0	15	70
59	Chlorthal dimethyl	GC	10.02	positive	300.9 → 166.9	55	300.9 → 222.9	25	70
60	Chrysene	GC	13.86	positive	228.0 → 226.0	40	228.0 → 227.0	25	70
61	Clindamycin	LC	5.33	positive	425.2 → 126.1	20	425.2 → 377.2	20	150
62	Clofentezine	LC	9.19	positive	303.1 → 138.0	12	303.1 → 102.0	40	120
63	Clothianidin	LC	3.91	positive	250.0 → 169.0	8	250.0 → 131.9	8	100
64	Cloxacillin	LC	6.86	positive	436.1 → 160.0	8	436.1 → 277.0	12	126
65	Coumachlor	LC	8.63	positive	343.1 → 162.8	15	342.1 → 285.0	15	120
66	Coumaphos	LC	8.98	positive	363.0 → 227.0	30	363.0 → 306.9	15	120
67	Coumatetralyl	LC	8.31	negative	291.1 → 141.0	30	291.1 → 247.0	20	140
68	Cyazofamid	LC	8.49	positive	325.0 → 108.0	20	325.0 → 261.1	15	90
69	Cyflufenamid	LC	9.18	positive	413.1 → 223.1	33	413.1 → 295.1	23	70
70	Cyfluthrin (sum of four isomers)	GC	16.07/16.19/16.25/16.32	positive	226.0 → 206.0	25	198.9 → 170.1	25	70
71	Cyhalothrin (lambda isomer)	GC	10.49	positive	181.1 → 152.1	10	181.1 → 127.1	46	70
72	Cymoxanil	LC	4.67	positive	199.1 → 128.0	4	199.1 → 110.9	12	90
73	Cypermethrin (sum of four isomers)	GC	16.34/16.44/16.52/16.63	positive	163.0 → 109.0	20	163.0 → 127.0	5	70
74	Cyproconazole (two isomers)	GC	11.98	positive	222.0 → 125.0	20	222.0 → 82.0	10	70
75	Cyprodinil	LC	8.46	positive	226.0 → 93.0	33	226.0 → 108	25	100
76	Cyromazine	LC	1.23	positive	167.1 → 85.0	16	167.1 → 125.0	20	120
77	Danofloxacin	LC	4.04	positive	358.2 → 340.1	20	358.2 → 82.1	50	159
78	Dazomet	GC	7.80	positive	161.9 → 44.0	28	161.9 → 89.0	5	70
79	Deltamethrin	LC	10.65	positive	523.0 → 281.0	10	523.0 → 506.0	5	100
80	Demeton-S-methyl	LC	5.97	positive	230.9 → 88.9	5	230.9 → 61.0	30	50
81	Demeton-S-methyl-sulfone (Dioxydemeton)	LC	3.31	positive	263.0 → 169.0	24	263.0 → 109.0	12	120
82	Dexamethasone	LC	7.16	positive	393.2 → 373.2	2	393.2 → 355.2	6	103
83	Diazinon	GC	8.29	positive	137.1 → 54.0	20	304.0 → 179.0	15	70
84	Dibenzo[a,h]anthracene	GC	19.15	positive	278.0 → 276.0	40	278.0 → 250.0	60	70
85	Dichlorodiphenyldichloroethane (p,p′ DDD)	GC	12.31	positive	235.0 → 165.0	20	235.0 → 199.0	15	70
86	Dichlorodiphenyldichloroethylene (p,p′ DDE)	GC	11.58	positive	318.0 → 176.0	60	318.0 → 248.0	30	70
87	Dichlorodiphenyltrichloroethane (p,p′ DDT)	GC	12.84	positive	235.0 → 165.0	40	235.0 → 199.0	15	70
88	Diclofenac	LC	8.73	positive	296.0 → 215.1	16	296.0 → 214.1	48	103
89	Dicloran	GC	7.80	positive	206.0 → 176.0	10	206.0 → 148.0	25	70
90	Diclorvos	GC	4.74	positive	184.9 → 93.0	10	185.0 → 109.0	15	70
91	Dicloxacillin	LC	7.24	positive	470.0 → 160.0	8	470.0 → 310.8	10	106
92	Dieldrin	GC	11.66	positive	263.0 → 228.0	15	277.0 → 241.0	15	70
93	Diethathyl ethyl	LC	8.71	positive	312.2 → 238.1	15	312.2 → 162.0	30	120
94	Diethofencarb	LC	7.57	positive	268.2 → 226.1	5	268.2 → 152.0	20	110
95	Difenacoum	LC	10.38	negative	443.2 → 135.0	40	443.2 → 293.0	35	200
96	Difenoconazole	LC	9.41	positive	406.1 → 250.9	28	406.1 → 337.0	16	176
97	Difethialone	LC	10.93	negative	537.3 → 79.0	50	537.3 → 151.0	45	220
98	Difloxacin	LC	3.86	positive	400.2 → 382.1	20	400.2 → 356.1	16	149
99	Diflubenzuron	LC	8.63	positive	311.0 → 158.0	8	311.0 → 141.0	32	90
100	Diflufenican	GC	13.27	positive	394.0 → 266.0	10	266.0 → 246.0	10	70
101	Dimethenamid-P (and its R-isomer)	LC	7.68	positive	276.1 → 244.1	10	276.1 → 168.1	20	125
102	Dimethoate	LC	4.21	positive	230.0 → 125.0	16	230.0 → 198.8	20	70
103	Dimethomorph (two isomers)	LC	7.86	positive	388.1 → 301.1	20	388.1 → 165.1	32	180
104	Dimethylphenylsulfamide (DMSA. metabolite of dichlofluanid)	LC	5.21	positive	201.1 → 92.1	15	201.1 → 137.1	5	100
105	Diniconazole-M	LC	9.34	positive	326.1 → 70.0	28	328.1 → 70.0	28	110
106	Dinocap	LC	10.51	negative	295.4 → 208.9	30	295.4 → 193.0	35	150
107	Diphacinone	LC	8.60	negative	339.1 → 167.0	25	339.1 → 145.0	20	170
108	Diphenylamine	GC	6.98	positive	168.0 → 167.2	15	169.0 → 66.0	15	70
109	Dodine	LC	9.02	positive	228.3 → 43.0	40	228.3 → 57.0	25	150
110	Doramectina	LC	11.31	positive	921.5 → 777.4	55	899.5 → 145.1	30	220
111	Endosulfan alfa	GC	11.21	positive	241.0 → 206.0	15	195.0 → 160.0	10	70
112	Endosulfan beta	GC	12.21	positive	241.0 → 206.0	15	195.0 → 159.0	15	70
113	Endosulfan sulfate	GC	12.96	positive	270.0 → 235.0	15	387.0 → 289.0	5	70
114	Endrin	GC	12.05	positive	263.0 → 193.0	35	245.0 → 173.0	25	70
115	Enrofloxacin	LC	3.94	positive	360.2 → 316.1	16	360.2 → 245.1	28	144
116	EPN	GC	13.90	positive	157.0 → 63.0	10	157.0 → 110.0	15	70
117	Epoxiconazole	LC	8.47	positive	330.0 → 120.9	24	330.1 → 100.9	50	120
118	Eprinomectin	LC	10.84	positive	878.5 → 186.0	15	936.5 → 490.4	60	160
119	Eritromicin	LC	6.74	positive	734.5 → 158.1	32	734.5 → 576.3	16	172
120	Esfenvalerate	GC	17.56	positive	167.1 → 125.1	15	167.1 → 89.1	45	70
121	Ethion (diethion)	LC	10.03	positive	385.0 → 199.0	5	385.0 → 171.0	10	100
122	Ethirimol	LC	4.80	positive	210.2 → 140.1	20	210.2 → 98.1	28	160
123	Ethofumesate	GC	9.59	positive	286.0 → 207.0	5	286.0 → 161.0	20	70
124	Ethoprophos	LC	8.38	positive	243.1 → 97.0	30	243.1 → 130.9	15	90
125	Etofenprox	GC	16.75	positive	163.0 → 107.0	20	163.0 → 135.0	10	70
126	Etoxazole	LC	10.34	positive	360.1 → 141.0	26	360.1 → 304.0	16	160
127	Famoxadone	LC	9.07	positive	392.1 → 330.9	5	392.2 → 238.1	12	110
128	Fenamidone	LC	9.06	positive	392.1 → 330.9	5	392.1 → 238.1	12	110
129	Fenamiphos	LC	7.72	positive	304.1 → 217.1	20	304.1 → 202.0	36	120
130	Fenamiphos sulfone	LC	8.63	positive	336.1 → 188.0	31	336.1 → 266.0	23	120
131	Fenamiphos sulfoxide	LC	5.93	positive	320.1 → 233.0	20	320.1 → 108.1	44	120
132	Fenarimol	GC	15.03	positive	139.0 → 75.0	30	139.0 → 111.0	15	70
133	Fenazaquin	LC	10.73	positive	307.2 → 57.1	25	307.2 → 161.1	16	90
134	Fenbendazole	LC	8.04	positive	300.1 → 268.1	20	300.1 → 159.0	36	156
135	Fenbuconazole	GC	16.17	positive	198.0 → 102.0	30	198.0 → 78.0	30	70
136	Fenhexamid	LC	8.35	positive	302.1 → 97.1	20	302.1 → 55.1	40	130
137	Fenitrothion	GC	9.57	positive	277.0 → 109.0	15	277.0 → 125.0	15	70
138	Fenoxycarb	LC	8.69	positive	302.1 → 88.0	20	302.1 → 116.1	10	110
139	Fenpropathrin	LC	10.43	positive	367.2 → 125.0	16	350.1 → 125.0	16	72
140	Fenpropidin	LC	7.13	positive	274.3 → 147.0	30	274.3 → 86.0	25	170
141	Fenpropimorph	LC	7.37	positive	304.3 → 147.1	30	304.3 → 130.0	25	120
142	Fenpyroximate	LC	10.49	positive	422.2 → 366.2	12	422.2 → 135.0	36	160
143	Fenthion	GC	8.90	positive	278.0 → 109.0	15	278.0 → 125.0	20	70
144	Fenthion oxon	LC	7.31	positive	263.1 → 231.2	16	263.1 → 216.0	24	120
145	Fenthion oxon sulfone	LC	4.50	positive	295.0 → 217.0	15	295.0 → 104.2	24	110
146	Fenthion oxon sulfoxide	LC	4.26	positive	279.0 → 264.2	20	279.0 → 104.1	28	110
147	Fenthion sulfone	LC	6.39	positive	311.0 → 125.0	22	311.0 → 109.0	28	140
148	Fenthion sulfoxide	LC	6.16	positive	295.0 → 108.9	30	295.0 → 280.0	18	140
149	Fenvalerate	GC	17.36	positive	167.0 → 125.1	22	167.0 → 89.0	30	70
150	Fipronil	LC	8.68	negative	435.0 → 330.0	12	435.0 → 249.9	26	116
151	Fipronil sulfide	GC	10.49	positive	351.0 → 255.0	20	420.0 → 351.0	25	70
152	Flocoumafen	LC	10.44	negative	541.3 → 382.0	25	541.3 → 161.0	40	230
153	Fluazinam	LC	10.01	negative	462.9 → 416.0	10	462.9 → 398.0	9	140
154	Flubendiamide	LC	8.82	positive	408.0 → 274.0	15	408.0 → 256.0	30	120
155	Flucythrinate (two isomers)	GC	16.67/16.84	positive	156.9 → 107.1	15	199.1 → 107.1	25	70
156	Fludioxonil	GC	11.51	positive	248.0 → 127.0	30	248.1 → 182.1	10	70
157	Flufenoxuron	LC	10.37	positive	489.1 → 158.0	20	489.1 → 140.9	56	110
158	Flumequine	LC	6.12	positive	262.1 → 244.0	16	262.1 → 202.0	32	116
159	Flunixin	LC	8.09	positive	297.1 → 279.1	24	297.1 → 264.1	32	141
160	Fluopyram	GC	10.61	positive	173.0 → 95.0	35	223.0 → 196.0	40	70
161	Fluoranthene	GC	10.66	positive	202.0 → 201.0	27	202.0 → 152.0	42	70
162	Fluorene	GC	6.81	positive	165.0 → 163.0	40	165.0 → 139.0	30	70
163	Fluquinconazole	GC	15.81	positive	340.0 → 298.0	15	340.0 → 286.0	25	70
164	Flusilazole	LC	8.64	positive	316.1 → 247.1	15	316.1 → 165.0	20	160
165	Flutolanil	LC	7.93	positive	324.1 → 262.1	16	324.1 → 242.1	24	130
166	Flutriafol	GC	11.26	positive	219.0 → 95.0	35	219.0 → 123.0	15	70
167	Fluvalinate tau	GC	17.56	positive	250.1 → 55.1	30	252.0 → 200.0	20	70
168	Fonofos	GC	8.24	positive	246.0 → 109.0	15	246.0 → 237.0	5	70
169	Formetanate	LC	1.76	positive	222.1 → 165.1	12	222.1 → 46.2	28	105
170	Fosthiazate	LC	6.50	positive	284.0 → 104.0	20	284.0 → 227.8	8	90
171	Heptachlor	GC	9.31	positive	272.0 → 237.0	15	274.0 → 239.0	15	70
172	Hexachlorobencene	GC	7.77	positive	284.0 → 214.0	40	284.0 → 249.0	25	70
173	Hexachlorocyclohexane (alpha)	GC	7.64	positive	219.0 → 109.0	10	219.0 → 183.0	10	70
174	Hexachlorocyclohexane (beta)	GC	8.02	positive	219.0 → 109.0	40	219.0 → 183.0	5	70
175	Hexachlorocyclohexane (delta)	GC	8.50	positive	219.0 → 109.0	45	219.0 → 183.0	5	70
176	Hexaclorocyclohexane (gamma. lindane)	GC	8.13	positive	291.0 → 109.0	40	219.0 →183.0	10	70
177	Hexaconazole (two isomers)	LC	8.49	positive	314.1 → 70.1	20	316.0 → 70.1	20	95
178	Hexaflumuron	LC	9.58	negative	458.8 → 439.0	8	458.8 → 175.0	30	100
179	Hexythiazox	LC	10.18	positive	353.1 → 227.9	8	353.1 → 168.1	24	120
180	Imazalil (enilconazole)	LC	6.53	positive	297.1 → 159.0	20	297.1 → 69.1	18	100
181	Imidacloprid	LC	3.93	positive	256.0 → 175.0	12	256.0 → 209.0	12	110
182	Indeno [1,2,3-cd] pyrene	GC	19.08	positive	276.0 → 274.0	50	276.0 → 272.0	60	70
183	Indoxacarb	LC	9.49	positive	528.1 → 293.1	10	528.1 → 202.8	48	140
184	Iprovalicarb	LC	8.18	positive	321.2 → 119.0	15	321.2 → 202.9	20	110
185	Isofenphos methyl	GC	10.38	positive	199.0 → 121.0	10	241.0 → 121.0	25	70
186	Isoprothiolane	LC	7.94	positive	291.1 → 189.0	30	291.1 → 145.0	36	100
187	Ivermectin B1a	LC	11.52	positive	897.5 → 753.5	50	897.5 → 329.3	60	160
188	Josamycin	LC	7.40	positive	860.5 → 173.9	40	860.5 → 108.9	40	200
189	Ketoprofen	LC	7.34	positive	255.1 → 209.1	8	255.1 → 77.1	48	123
190	Kresoxim methyl	GC	11.78	positive	116.0 → 89.0	15	206.0 → 131.0	10	70
191	Levamisole	LC	3.12	positive	205.1 → 178.1	20	205.1 → 123.0	32	141
192	Lincomycin	LC	3.50	positive	407.2 → 126.1	24	407.2 → 359.2	16	150
193	Linuron	LC	7.54	positive	249.0 → 160.1	20	249.0 → 182.3	8	120
194	Lufenuron	LC	10.05	negative	509.0 → 339.0	5	509.0 → 326.1	15	90
195	Mandipropamid	LC	7.90	positive	412.1 → 328.1	8	412.1 → 356.1	4	130
196	Mebendazole	LC	6.68	positive	296.1 → 264.1	20	296.1 → 77.0	48	151
197	Mefenamic acid	LC	9.52	positive	242.1 → 209.1	28	242.1 → 180.1	0	108
198	Mefenoxam (metalaxyl-M)	LC	6.95	positive	280.0 → 220.0	10	280.0 → 192.0	15	110
199	Meloxicam	LC	7.17	positive	352.5 → 114.8	20	352.5 → 140.8	20	130
200	Mepanipyrim	GC	11.13	positive	222.0 → 221.0	15	222.0 → 207.0	15	70
201	Mepiquat	LC	0.64	positive	114.0 → 98.0	36	114.0 → 70.0	45	100
202	Metaflumizone	LC	9.94	negative	505.0 → 302.0	14	541.0 → 302.0	20	90
203	Metalaxyl	GC	9.31	positive	234.0 → 146.1	20	249.0 → 146.0	20	70
204	Metaldehyde	LC	3.87	positive	194.1 → 61.9	5	194.1 → 106.0	5	50
205	Metconazole	LC	9.17	positive	320.1 → 70.2	33	322.1 → 70.2	24	250
206	Methamidophos (two isomers)	LC	1.18	positive	142.0 → 94.0	12	142.0 → 125.0	12	85
207	Methidathion	LC	7.12	positive	320.1 → 144.8	8	320.1 → 85.0	30	84
208	Methiocarb	LC	7.67	positive	226.1 → 169.0	4	226.1 → 121.1	12	90
209	Methiocarb-sufone	LC	4.52	positive	258.1 → 201.1	8	258.1 → 122.1	22	100
210	Methiocarb-sulfoxide	LC	4.03	positive	242.0 → 185.0	22	242.0 → 122.0	28	90
211	Methomyl	LC	3.23	positive	163.1 → 88.0	5	163.0 → 106.0	8	80
212	Methoxyfenozide	LC	8.00	positive	369.2 → 149.0	10	369.2 → 313.1	15	85
213	Metoxychlor	GC	13.98	positive	227.0 → 141.0	20	227.0 → 169.0	15	70
214	Metrafenone	LC	9.27	positive	409.1 → 209.1	8	411.1 → 209.1	12	108
215	Metronidazole	LC	2.63	positive	172.1 → 128.0	12	172.1 → 82.1	24	98
216	Mevinphos (phosdrin)	LC	4.38	positive	225.0 → 193.1	15	225.0 → 127.0	12	65
217	Mirex	GC	5.66	positive	237.0 → 143.0	30	274.0 → 237.0	10	70
218	Monocrotophos	LC	3.31	positive	224.1 → 126.8	12	224.1 → 98.1	15	100
219	Moxidectin	LC	11.24	positive	641.4 → 529.2	5	641.4 → 499.2	5	100
220	Myclobutanil	LC	8.10	positive	289.1 → 70.1	16	289.1 → 125.1	32	110
221	N-(2,4-dimethylphenyl)-N′-methylformamidine (DMPF, metabolite of amitraz)	LC	3.35	positive	163.1 → 122.1	15	163.1 → 107.1	15	100
222	N,N-Dimethyl-N′-p-tolylsulphamide (DMST, metabolite of tolyfluanid)	LC	6.06	positive	215.1 → 106.1	10	215.1 → 151.1	4	90
223	Nafcillin	LC	7.33	positive	415.0 → 199.1	8	415.0 → 171.0	36	103
224	Naphtalene	GC	4.45	positive	128.0 → 127.0	15	128.0 → 102.0	25	70
225	Naproxen	LC	7.59	positive	231.0 → 185.0	10	231.1 → 169.9	21	120
226	Nitenpyram	LC	3.30	positive	271.1 → 56.1	36	271.1 → 224.9	12	100
227	Novobiocin	LC	9.69	positive	613.2 → 218.1	10	613.2 → 396.1	10	150
228	Nuarimol	GC	13.27	positive	235.0 → 139.0	15	235.0 → 111.0	40	70
229	Ofurace	LC	5.97	positive	282.0 → 159.9	20	282.0 → 147.9	30	100
230	Omethoate	LC	2.80	positive	214.1 → 124.8	22	214.1 → 183.0	5	100
231	Oxadixyl	LC	5.43	positive	279.1 → 219.2	5	279.1 → 132.2	32	110
232	Oxamyl	LC	2.87	positive	237.1 → 72.0	12	237.1 → 90.0	5	70
233	Oxamyl-oxime	LC	2.46	positive	163.3 → 115.2	10	163.3 → 72.1	10	70
234	Oxfendazole	LC	5.61	positive	316.1 → 159.0	32	316.1 → 191.1	16	166
235	Oxolinic acid	LC	5.04	positive	262.1 → 216.0	32	262.1 → 160.0	36	110
236	Oxydemeton methyl	LC	3.01	positive	247.0 → 169.0	12	247.0 → 109.0	24	100
237	Oxyfluorfen	GC	11.68	positive	252.0 → 146.0	40	300.0 → 223.0	15	70
238	Paclobutrazol	LC	7.89	positive	294.1 → 70.1	16	294.1 → 125.2	36	115
239	Parathion methyl	GC	9.12	positive	263.0 → 109.0	15	263.0 → 79.0	30	70
240	PCB 28	GC	9.01	positive	256.0 → 186.0	25	256.0 → 151.0	50	70
241	PCB 52	GC	9.58	positive	292.0 → 222.0	25	292.0 → 220.0	25	70
242	PCB 77	GC	11.73	positive	292.0 → 220.0	25	292.0 → 222.0	25	70
243	PCB 81	GC	11.56	positive	292.0 → 220.0	25	292.0 → 222.0	25	70
244	PCB 101	GC	11.08	positive	326.0 → 256.0	30	328.0 → 256.0	30	70
245	PCB 105	GC	12.66	positive	326.0 → 256.0	30	328.0 → 256.0	30	70
246	PCB 114	GC	12.38	positive	326.0 → 256.0	30	328.0 → 256.0	30	70
247	PCB 118	GC	12.18	positive	326.0 → 256.0	30	328.0 → 256.0	30	70
248	PCB 123	GC	12.10	positive	326.0 → 256.0	30	328.0 → 256.0	30	70
249	PCB 126	GC	13.23	positive	326.0 → 256.0	30	328.0 → 256.0	30	70
250	PCB 138	GC	13.07	positive	360.0 → 290.0	25	360.0 → 288.0	25	70
251	PCB 153	GC	12.57	positive	360.0 → 290.0	25	360.0 → 288.0	25	70
252	PCB 156	GC	13.96	positive	360.0 → 290.0	25	360.0 → 288.0	25	70
253	PCB 157	GC	14.07	positive	360.0 → 290.0	25	360.0 → 288.0	25	70
254	PCB 167	GC	13.55	positive	360.0 → 290.0	25	360.0 → 288.0	25	70
255	PCB 169	GC	14.61	positive	360.0 → 290.0	25	360.0 → 288.0	25	70
256	PCB 180	GC	14.25	positive	394.0 → 324.0	30	394.0 → 322.0	30	70
257	PCB 189	GC	15.25	positive	394.0 → 324.0	30	394.0 → 322.0	30	70
258	Penconazole	GC	10.52	positive	248.0 → 157.0	30	248.0 → 192.0	15	70
259	Pencycuron	LC	9.33	positive	329.1 → 125.1	24	329.1 → 217.9	12	160
260	Pendimethalin	GC	10.49	positive	252.0 → 162.0	10	252.0 → 191.0	5	70
261	Penicillin V	LC	6.47	positive	383.2 → 159.9	10	383.2 → 113.9	40	130
262	Permethrin	GC	15.69	positive	183.0 → 128.0	15	183.1 → 153.1	15	70
263	Phenanthrene	GC	8.40	positive	178.0 → 176.0	35	178.0 → 152.0	28	70
264	Phenylbutazone	LC	8.25	positive	309.2 → 160.2	20	309.2 → 77.1	55	140
265	Phosalone	LC	9.20	positive	385.1 → 182.0	20	385.1 → 110.9	55	80
266	Phosmet	LC	7.34	positive	318.0 → 159.9	16	318.0 → 133.0	40	90
267	Pthalamide (Folpet deg)	GC	5.94	positive	104.0 → 50.0	25	147.0 → 76.0	25	70
268	Pirimicarb	LC	5.11	positive	239.1 → 72.1	20	239.1 → 182.1	12	100
269	Pirimicarb-desmethyl	LC	3.71	positive	225.1 → 168.1	8	225.1 → 72.1	20	100
270	Pirimiphos ethyl	GC	10.26	positive	318.0 → 166.0	15	318.0 → 182.0	15	70
271	Pirimiphos methyl	LC	9.13	positive	306.1 → 164.0	20	306.1 → 108.1	32	100
272	Prochloraz	LC	9.08	positive	376.0 → 308.0	10	376.0 → 70.1	20	100
273	Procymidone	GC	10.80	positive	283.0 → 67.0	40	283.0 → 68.0	25	70
274	Profenofos	LC	9.75	positive	375.0 → 305.0	20	373.0 → 303.0	20	100
275	Propamocarb	LC	2.85	positive	189.2 → 102.0	12	189.2 → 144.0	8	110
276	Propargite	LC	10.37	positive	368.2 → 231.1	4	368.2 → 175.0	12	88
277	Propiconazole	LC	9.01	positive	342.0 → 69.0	21	342.0 → 159.0	39	90
278	Propoxur	LC	5.83	positive	210.1 → 168.1	35	210.1 → 65.1	40	70
279	Propyzamide (pronamide)	LC	7.92	positive	256.1 → 190.0	16	256.1 → 173.0	25	90
280	Proquinazid	GC	13.32	positive	288.0 → 245.0	15	288.0 → 217.0	30	70
281	Prothioconazol	GC	11.85	positive	186.0 → 49.0	20	186.0 → 53.0	25	70
282	Prothiophos	GC	11.45	positive	266.9 → 221.0	35	162.0 → 63.1	30	70
283	Pymetrozine	LC	2.74	positive	218.1 → 105.0	20	218.1 → 78.0	52	120
284	Pyraclostrobin	LC	9.15	positive	388.1 → 193.8	8	388.1 → 163.1	28	120
285	Pyrazophos	LC	9.22	positive	374.1 → 222.1	23	374.1 → 194.0	32	100
286	Pyrene	GC	11.13	positive	202.0 → 201.0	27	202.0 → 200.0	45	70
287	Pyridaben	LC	10.75	positive	365.2 → 309.0	8	309.1 → 147.0	16	168
288	Pyridaphenthion	LC	8.11	positive	341.0 → 189.0	22	341.0 → 205.0	34	100
289	Pyrimethanil	GC	8.27	positive	198.0 → 118.0	40	198.0 → 158.0	20	70
290	Pyriproxifen	LC	10.07	positive	322.2 → 96.0	12	322.2 → 184.9	24	80
291	Quinalfos	LC	8.72	positive	299.1 → 96.9	30	299.1 →147.1	20	130
292	Quinoxyfen	LC	10.13	positive	308.0 → 197.0	32	308.2 → 161.8	55	120
293	Rifampicin	LC	7.89	positive	823.5 → 791.4	15	823.5 → 399.1	25	160
294	Rotenone	LC	8.64	positive	395.1 → 213.1	20	395.1 → 192.1	25	150
295	Roxithromycin	LC	7.67	positive	838.5 → 158.1	40	838.5 → 116.1	55	200
296	Sarafloxacin	LC	4.16	positive	386.1 → 342.1	16	386.1 → 299.1	28	144
297	Simazine	LC	5.81	positive	202.4 → 68.1	30	202.4 → 68.1	20	120
298	Spinosad (two isomers)	LC	9.10/9.43	positive	732.4 → 142.0	22	732.4 → 98.0	60	130
299	Spiramycin (two isomers)	LC	4.58/4.90	positive	439.1 → 101.1	20	439.1 → 88.0	50	70
300	Spirodiclofen	LC	10.50	positive	411.1 → 71.2	15	411.1 → 313.0	5	110
301	Spiromesifen	LC	10.27	positive	388.0 → 273.0	25	273.0 → 187.0	15	110
302	Spirotetramat	LC	8.23	positive	374.2 → 302.2	12	374.2 → 216.1	36	150
303	Spiroxamine	LC	7.55	positive	298.3 → 144.1	16	298.3 → 100.1	32	120
304	Strychnine	LC	3.00/3.61	positive	335.1 → 184.0	45	335.1 → 156.0	40	105
305	Sulfacetamide	LC	2.13	positive	215.3 → 155.9	10	215.3 → 92.0	20	90
306	Sulfachloropiridacine	LC	3.77	positive	285.0 → 156.0	12	285.0 → 92.1	28	101
307	Sulfadiacine	LC	2.80	positive	251.0 → 92.0	28	251.0 → 156.0	12	111
308	Sulfadimetoxine	LC	4.81	positive	311.0 → 92.0	32	311.0 → 156.0	16	139
309	Sulfadoxine	LC	4.12	positive	311.1 → 92.0	32	311.1 → 156.0	16	126
310	Sulfameracine	LC	3.26	positive	265.0 → 92.0	28	265.0 → 156.0	12	126
311	Sulfametacine	LC	3.44	positive	279.1 → 186.0	12	279.1 → 92.0	32	134
312	Sulfametizole	LC	3.37	positive	271.0 → 92.0	28	271.0 → 155.9	8	103
313	Sulfametoxazole	LC	3.93	positive	254.0 → 92.0	28	254.0 → 156.0	12	111
314	Sulfametoxipiridacine	LC	3.45	positive	281.0 → 155.9	12	281.0 → 92.1	28	121
315	Sulfamonomethoxine	LC	4.11	positive	281.1 → 156.0	14	281.1 → 92.1	32	120
316	Sulfapyridine	LC	2.82	positive	250.0 → 156.0	12	250.0 → 92.0	28	126
317	Sulfaquinoxaline	LC	4.99	positive	301.0 → 156.0	12	301.0 → 92.1	32	159
318	Sulfatiazole	LC	2.98	positive	256.0 → 92.0	28	256.0 → 156.0	12	106
319	Sulfisoxazole	LC	4.12	positive	268.0 → 156.0	8	268.0 → 92.1	24	106
320	Tebuconazole	LC	8.92	positive	308.2 → 70.2	22	308.2 → 125.1	53	120
321	Tebufenocide	LC	8.66	positive	353.1 → 132.9	22	353.1 → 297.1	20	90
322	Tebufenpyrad	LC	9.88	positive	334.2 → 117.0	47	334.2 → 145.0	37	180
323	Teflubenzuron	LC	10.01	negative	379.0 → 339.0	15	379.0 → 196.0	25	100
324	Tefluthrin	GC	8.42	positive	177.0 → 127.0	15	177.0 → 87.0	15	70
325	Telodrin (isobenzan)	GC	10.14	positive	310.8 → 240.8	25	310.8 → 274.8	5	70
326	Terbufos	GC	8.15	positive	231.0 → 97.0	20	231.0 → 129.0	15	70
327	Terbuthylazine	GC	8.12	positive	214.0 → 104.0	20	214.0 → 132.0	10	70
328	Tetrachlorvinphos	LC	8.72	positive	367.0 → 127.0	16	365.0 → 127.0	16	110
329	Tetraconazole	GC	10.04	positive	336.0 → 204.0	35	336.0 → 218.0	20	70
330	Tetradifon	GC	14.36	positive	158.9 → 111.0	20	354.0 → 159.0	10	70
331	Tetramethrin	GC	13.87	positive	164.0 → 77.0	30	164.0 → 107.0	15	70
332	Thiabendazole	GC	5.94	positive	201.0 → 174.0	15	201.0 → 130.0	30	70
333	Thiacloprid	LC	4.80	positive	253.0 → 126.0	16	253.0 → 90.0	40	140
334	Thiamethoxam	LC	3.59	positive	292.0 → 211.1	8	292.0 → 132.0	22	80
335	Thiophanate methyl	LC	5.87	positive	343.0 → 151.0	20	343.0 → 93.0	46	90
336	Tolclofos methyl	GC	9.21	positive	265.0 → 93.0	30	265.0 → 220.0	25	70
337	Tolfenamic acid	LC	9.80	negative	260.0 → 216.1	8	260.0 → 35.1	20	108
338	Triadimefon	LC	8.03	positive	294.1 → 69.3	20	294.1 → 197.2	15	100
339	Triadimenol	LC	8.22	positive	296.1 → 70.0	10	298.1 → 70.0	10	80
340	Triazophos (hostathion)	LC	8.18	positive	314.1 → 162.0	19	314.1 → 118.9	35	100
341	Trifloxystrobin	LC	9.50	positive	409.1 → 186.0	12	409.1 → 145.0	52	110
342	Triflumizole	LC	9.53	positive	346.1 → 278.0	4	345.9 → 73.0	15	80
343	Triflumuron	LC	9.19	positive	359.0 → 156.0	8	359.0 → 139.0	32	120
344	Trifluralin	GC	7.27	positive	264.0 → 160.0	15	306.0 → 264.0	5	70
345	Trimethoprim	LC	3.45	positive	291.2 → 123.0	24	291.2 → 230.1	20	162
346	Triticonazole	LC	8.38	positive	318.1 → 70.1	33	320.1 → 70.1	16	110
347	Tylmicosin	LC	5.52	positive	869.6 → 174.1	48	869.6 → 696.4	44	294
348	Tylosin	LC	6.76	positive	916.5 → 174.1	40	916.5 → 772.4	28	210
349	Vinclozolin	GC	9.10	positive	212.0 → 145.0	25	212.0 → 109.0	50	70
350	Warfarin	LC	7.86	negative	307.1 → 161.1	20	307.1 → 250.1	20	140
351	Zoxamide	LC	9.03	positive	336.0 → 187.1	25	187.1 → 88.9	40	200

## Appendix B

**Table A2:** Method validation results: Limits of quantification (LOQ), percentage recoveries and relative standard deviation obtained from intraday and interday studies.

			0.4 ng/mL			1 ng/mL			4 ng/mL			20 ng/mL			40 ng/mL		
				Precision (RSD. %)			Precision (RSD. %)			Precision (RSD. %)			Precision (RSD. %)			Precision (RSD. %)	
No.	Compound	LOQ (ng/mL)	Rec (%)	Intraday	Interday	Rec. (%)	Intraday	Interday	Rec. (%)	Intraday	Interday	Rec. (%)	Intraday	Interday	Rec. (%)	Intraday	Interday
1	2-Phenylphenol	2				101.43	19.69	18.85	107.17	20.66	19.28	106.47	18.24	17.13	96.41	13.81	14.32
2	4,4’-Dichlorobenzophenone (metabolite of dicofol)	2				91.36	13.23	14.48	86.99	13.79	15.85	116.77	11.76	10.07	98.37	15.59	15.85
3	Abamectine	4							106.43	14.53	13.65	93.89	5.21	5.55	99.43	14.50	14.58
4	Acenaphthene	1.2				110.88	19.39	19.29	94.91	6.78	7.14	96.45	12.08	12.52	95.23	4.52	4.75
5	Acenaphtylene	2				107.06	18.90	21.39	95.74	12.53	13.09	100.50	12.26	12.20	98.40	2.93	2.98
6	Acephate	8										90.75	20.15	22.20	93.24	14.96	16.04
7	Acetamiprid	2				122.13	8.03	6.57	101.26	14.21	14.03	104.42	4.70	4.50	104.71	4.76	4.55
8	Acrinathrin	4							116.08	16.73	14.41	103.11	22.19	21.52	104.55	5.45	5.21
9	Albendazole	0.4	121.03	11.39	9.41	92.56	6.35	6.86	86.92	12.48	14.36	99.53	3.66	3.68	105.10	4.30	4.09
10	Aldicarb	0.8	119.20	13.25	10.26	97.22	7.43	7.64	89.92	14.70	16.35	100.15	4.24	4.23	107.89	5.24	4.86
11	Aldicarb-sulfone	2				121.84	18.51	15.19	94.84	16.90	19.91	85.77	12.91	15.05	97.26	10.13	10.42
12	Aldicarb-sulfoxide	4							104.82	18.42	17.11	81.91	5.97	7.29	105.26	12.19	11.58
13	Aldrin	2				123.66	19.11	15.45	91.59	15.25	16.65	106.54	5.58	5.24	97.33	12.12	12.45
14	Anthracene	1.6				99.67	15.35	15.40	91.47	15.47	16.91	105.34	7.32	6.95	94.68	6.50	6.87
15	Atrazine	0.8	124.53	4.21	3.38	93.04	7.01	7.53	87.60	13.23	15.10	102.49	5.46	5.33	110.16	6.62	6.01
16	Azinphos-methyl	2				126.85	18.74	14.77	103.98	13.75	13.22	105.19	3.50	3.33	102.89	5.44	5.29
17	Azoxystrobin	0.4	116.28	7.94	6.83	95.41	3.96	4.15	92.31	6.03	6.53	102.95	4.52	4.39	101.82	2.11	2.07
18	BDE-28	1.2				100.53	14.19	14.12	81.65	9.78	11.98	104.45	6.20	5.94	96.62	11.51	11.91
19	BDE-47	0.8	98.71	20.31	20.58	109.45	21.87	19.98	89.88	8.16	9.08	110.24	8.74	7.93	95.96	8.89	9.26
20	BDE-85	0.8	100.77	15.79	15.59	103.10	16.65	16.15	85.61	12.99	15.17	108.23	16.09	14.87	98.51	17.91	13.96
21	BDE-99	0.4	107.54	18.55	19.69	103.74	15.02	14.48	89.65	12.66	14.12	114.05	18.00	15.78	90.97	11.97	13.16
22	BDE-100	0.8	98.15	21.94	22.35	106.72	17.54	21.81	93.71	10.60	11.31	109.98	12.80	11.64	94.05	19.80	21.05
23	BDE-153	0.8	93.58	16.95	19.48	108.66	17.04	19.88	93.65	15.26	16.29	111.40	15.44	13.86	96.11	17.16	17.85
24	BDE-154	0.4	96.19	15.79	16.42	113.57	19.84	17.47	92.43	10.71	11.59	118.15	16.34	13.83	93.42	13.93	15.62
25	BDE-183	4							78.46	5.92	7.55	104.68	12.90	12.32	95.99	8.73	9.09
26	Benalaxyl	0.4	116.67	15.06	12.91	94.56	3.53	3.73	97.64	6.31	6.46	101.10	3.37	3.33	101.39	3.01	2.97
27	Bendiocarb	0.8	123.80	9.46	7.29	90.11	14.65	16.26	93.23	14.26	15.30	101.72	3.10	3.05	110.76	8.93	8.06
28	Bendiocarb metabolite (2,2-dimethylbenzo-1. 3-dioxol-4-ol)	4							86.86	16.14	21.61	102.82	16.72	16.26	95.65	3.05	3.19
29	Benfuracarb	0.8	121.43	6.42	5.24	88.09	12.05	13.68	85.50	9.48	11.09	99.93	4.45	4.45	102.76	5.96	5.80
30	Benzo[a]anthracene	0.8	98.90	19.38	19.71	97.89	8.00	8.17	85.04	4.75	5.59	109.73	14.44	13.16	101.08	11.10	10.98
31	Benzo[a]pyrene	0.8	118.01	19.78	20.66	94.10	10.74	11.41	90.35	9.53	10.55	106.01	11.75	11.08	98.16	14.17	14.44
32	Benzo[b]fluoranthene	1.2				101.28	11.89	11.61	91.62	10.11	11.03	105.11	8.02	7.63	93.83	8.80	9.38
33	Benzo[ghi]perylene	0.8	80.16	19.32	20.41	100.35	15.43	15.38	93.47	9.37	10.02	114.39	6.35	5.55	95.50	10.80	11.31
34	Benzo[k]fluoranthene	1.2				102.66	13.24	12.90	88.84	14.63	16.47	101.90	7.87	7.72	99.44	17.32	17.42
35	Bifenthrin	2				118.63	18.93	19.10	86.17	14.08	16.34	113.53	16.83	14.82	98.99	10.04	10.14
36	Bitertanol	0.4	121.41	23.34	18.61	84.35	14.31	16.97	80.69	10.74	13.31	99.71	4.76	4.77	105.11	2.05	1.95
37	Boscalid (formerly nicobifen)	0.8	74.96	22.43	22.81	85.76	5.16	6.02	85.67	9.78	11.42	112.15	6.66	5.94	89.33	8.33	9.32
38	Brodifacoum	0.4	102.44	23.57	23.01	88.99	13.67	15.36	85.84	12.99	15.13	90.41	5.61	6.21	107.51	6.87	6.39
39	Bromadiolone	0.4	118.13	11.13	19.82	97.11	20.55	19.19	89.46	13.13	14.68	97.66	8.85	9.06	101.30	8.87	8.76
40	Bromopropylate	0.4	110.83	21.15	22.86	96.37	19.85	20.60	93.29	8.21	8.80	107.25	10.02	9.34	96.53	10.67	11.05
41	Bromuconazole (two isomers)	1.6				106.70	16.63	14.96	89.48	17.19	19.21	112.75	13.08	11.60	94.54	8.05	8.51
42	Bupirimate	1.2				86.77	10.74	12.38	93.94	5.74	6.11	98.53	6.03	6.12	102.43	4.99	4.87
43	Buprofezin	0.4	115.89	11.18	9.65	93.22	1.92	2.06	88.57	10.64	12.01	101.63	4.50	4.43	102.68	3.54	3.45
44	Cadusafos (ebufos)	0.4	126.81	3.48	2.74	94.26	4.04	4.29	96.81	5.74	5.93	101.63	5.00	4.92	100.58	3.04	3.02
45	Carbaryl	0.8	118.84	13.56	10.52	96.23	6.27	6.52	87.81	11.26	12.82	100.43	4.37	4.35	108.80	4.34	3.99
46	Carbendazim (azole)	2				120.17	8.97	6.40	98.20	9.83	10.01	100.39	2.72	2.71	103.56	3.81	3.68
47	Carbofuran	0.4	123.88	10.74	8.67	94.30	6.42	6.81	88.14	13.54	15.36	104.41	6.50	6.23	105.57	3.33	3.15
48	Carbofuran-3-hydroxy	0.8	120.98	10.76	8.89	89.04	8.10	9.10	94.46	15.41	16.31	100.86	3.61	3.58	107.55	4.88	4.54
49	Carbosulfan	1.2				109.16	14.87	19.19	89.29	21.22	20.62	92.19	22.12	23.99	96.57	21.02	21.77
50	Cefuroxima axetil (two isomers)	4							132.33	13.51	10.21	119.80	8.73	7.29	127.08	13.98	11.00
51	Chloramphenicol	16													98.91	7.74	7.83
52	Chlorantraniliprole	16													106.00	2.93	2.76
53	Chlorfenvinphos	0.8	114.28	16.10	14.09	97.14	14.61	15.04	91.56	15.96	17.43	104.40	2.64	2.53	106.32	3.78	3.56
54	Chlorobenzilate	1.6				110.47	17.93	16.23	89.89	4.22	4.69	114.60	12.66	11.05	90.71	2.94	3.24
55	Chlorophacinone	8										98.40	21.84	22.36	99.28	8.31	8.37
56	Chlorpropham	2				119.39	17.61	13.13	93.11	14.39	15.45	112.93	6.10	5.40	100.11	21.56	21.54
57	Chlorpyrifos	1.6				74.81	17.17	19.32	92.21	1.74	1.89	119.54	9.95	8.32	90.50	6.87	7.59
58	Chlorpyrifos methyl	2				115.24	17.63	14.98	88.08	15.56	19.02	90.85	9.97	10.97	106.30	6.98	6.57
59	Chlorthal dimethyl	0.8	104.38	17.25	19.43	96.72	14.55	15.04	91.37	6.53	7.15	113.90	9.82	8.62	90.34	3.66	4.05
60	Chrysene	1.6				109.91	12.42	11.30	91.83	7.03	7.66	106.10	17.06	16.08	100.25	11.34	11.31
61	Clindamycin	4							129.25	10.53	8.15	109.20	7.44	6.81	107.36	6.37	5.93
62	Clofentezine	0.8	116.80	20.59	17.63	94.99	7.99	8.41	83.66	11.18	13.36	96.74	7.45	7.70	105.68	4.56	4.31
63	Clothianidin	12										105.52	8.63	8.18	97.88	11.44	11.69
64	Cloxacillin	8										99.33	18.27	18.39	107.79	10.04	9.31
65	Coumachlor	0.8	120.85	16.85	20.84	99.90	12.14	12.15	85.48	16.06	18.79	101.57	4.20	4.14	102.89	2.20	2.14
66	Coumaphos	0.8	112.59	14.03	12.46	86.24	15.34	17.79	86.49	10.71	12.38	105.36	4.20	3.99	103.38	4.36	4.22
67	Coumatetralyl	1.6				116.12	22.62	19.48	97.65	9.98	10.22	96.87	4.29	4.43	102.71	4.39	4.27
68	Cyazofamid	2				112.26	13.66	12.17	100.29	12.06	12.03	103.98	7.08	6.81	97.87	5.04	5.15
69	Cyflufenamid	1.6				116.75	17.97	23.96	96.02	19.44	20.25	98.16	6.89	7.02	105.14	7.86	7.48
70	Cyfluthrin (sum of four isomers)	8										128.33	19.82	15.44	87.94	19.51	21.60
71	Cyhalothrin (lambda isomer)	4							118.45	17.58	14.84	110.36	18.54	16.80	102.15	14.52	14.21
72	Cymoxanil	2				127.98	23.20	18.13	112.78	13.14	11.65	111.18	3.17	2.85	107.24	7.19	6.70
73	Cypermethrin (sum of four isomers)	20										125.42	15.64	12.47	74.31	10.18	13.70
74	Cyproconazole (two isomers)	4				120.66	19.73	20.01	89.98	15.51	17.24	110.68	5.93	5.36	93.41	7.33	7.85
75	Cyprodinil	1.2				110.91	13.69	12.34	89.72	15.25	17.00	97.14	8.22	8.46	104.22	7.41	7.11
76	Cyromazine	8										94.58	11.29	11.94	96.74	11.32	11.70
77	Danofloxacin	8										113.55	18.30	16.12	89.76	10.39	11.58
78	Dazomet	4							71.53	17.55	21.50	83.03	13.84	16.67	90.82	16.95	18.66
79	Deltamethrin	4							95.89	17.74	19.36	107.28	18.61	17.24	100.61	12.28	12.21
80	Demeton-S-methyl	0.8	123.35	9.93	7.74	95.67	3.32	3.47	87.38	12.64	14.47	101.00	5.18	5.13	108.47	6.18	5.70
81	Demeton-S-methyl-sulfone (Dioxydemeton)	12										80.24	4.83	6.02	96.53	6.34	6.57
82	Dexamethasone	2				123.79	17.67	12.29	104.61	13.93	13.32	106.53	14.48	13.59	102.96	4.32	4.20
83	Diazinon	1.2				101.78	19.05	18.68	97.76	8.04	8.22	110.63	15.03	13.59	92.16	7.39	8.02
84	Dibenzo[a.h]anthracene	0.8	70.39	18.68	16.54	112.50	16.62	14.77	93.06	11.73	12.60	111.29	4.83	4.34	84.41	6.54	7.75
85	Dichlorodiphenyldichloroethane (p,p′ DDD)	0.8	88.27	19.42	17.67	112.25	6.36	5.67	92.57	11.03	11.92	101.42	6.07	5.99	93.46	4.77	5.10
86	Dichlorodiphenyldichloroethylene (p,p′ DDE)	0.8	99.71	18.39	18.44	110.56	17.85	18.67	97.62	7.46	7.64	104.29	0.72	0.69	98.40	8.58	8.72
87	Dichlorodiphenyltrichloroethane (p,p′ DDT)	2				101.87	15.98	14.95	104.65	20.63	19.71	110.43	6.32	5.72	92.27	6.86	7.43
88	Diclofenac	4							111.25	21.71	19.51	91.03	12.48	13.71	94.44	12.42	13.15
89	Dicloran	4							95.68	14.38	15.03	113.17	15.89	14.04	92.28	5.09	5.52
90	Diclorvos	8										113.48	10.38	9.15	98.10	2.98	3.04
91	Dicloxacillin	12										77.54	12.35	15.93	103.35	10.63	10.29
92	Dieldrin	8										80.70	18.40	18.43	95.08	5.85	6.15
93	Diethathyl ethyl	0.4	113.26	12.83	20.16	96.15	13.17	13.70	89.84	15.24	16.96	97.95	6.16	6.29	99.30	7.45	7.50
94	Diethofencarb	0.4	113.98	6.38	5.60	90.65	3.94	4.35	90.80	10.90	12.00	101.24	1.48	1.46	104.26	1.97	1.89
95	Difenacoum	0.8	117.66	13.84	21.51	88.27	10.36	11.74	85.40	7.72	9.04	92.19	6.14	6.66	104.37	3.77	3.61
96	Difenoconazole	0.8	119.78	17.98	13.85	94.45	8.83	9.35	84.08	7.72	9.18	97.08	6.45	6.64	106.47	4.12	3.87
97	Difethialone	1.6				112.44	10.79	9.60	88.10	18.22	20.68	95.86	6.45	6.73	103.22	8.66	8.39
98	Difloxacin	4							123.36	19.34	15.68	102.87	8.83	8.58	99.17	8.54	8.61
99	Diflubenzuron	1.6				121.03	16.61	18.98	99.73	16.54	16.58	102.86	17.01	16.54	105.63	4.13	3.91
100	Diflufenican	0.4	114.39	18.24	19.49	101.56	9.38	9.24	95.89	9.59	10.00	115.49	8.35	7.23	95.62	9.05	9.46
101	Dimethenamid-P (and its R-isomer)	0.4	112.73	15.63	12.74	87.82	4.74	5.40	90.21	8.85	9.81	103.64	3.95	3.81	102.49	5.02	4.90
102	Dimethoate	0.8	121.83	20.22	15.57	93.11	5.47	5.87	87.05	11.89	13.66	100.07	10.40	10.39	108.51	7.39	6.81
103	Dimethomorph (two isomers)	0.4	123.37	18.68	15.14	87.72	6.32	7.20	87.24	11.29	12.94	104.93	4.63	4.41	105.80	5.87	5.55
104	Dimethylphenylsulfamide (DMSA, metabolite of dichlofluanid)	4							114.05	11.55	10.13	109.55	9.65	8.81	102.37	4.33	4.23
105	Diniconazole-M	1.2				97.80	16.52	16.89	88.78	6.85	7.72	102.75	14.54	14.15	100.66	5.86	5.82
106	Dinocap	4							115.19	16.40	14.24	105.54	2.90	2.75	105.88	5.85	5.53
107	Diphacinone	8										96.74	18.11	18.72	103.66	19.59	18.90
108	Diphenylamine	1.6				92.81	20.64	13.01	96.18	5.32	5.53	106.11	10.45	9.85	93.91	2.44	2.60
109	Dodine	0.8	107.19	12.63	11.78	97.64	8.18	8.38	87.26	10.47	12.00	102.27	5.32	5.20	102.46	4.26	4.16
110	Doramectina	8										101.04	19.63	19.43	99.13	9.03	9.11
111	Endosulfan alfa	2				112.45	11.73	10.43	84.22	19.47	23.12	122.57	20.34	20.75	96.35	10.24	10.63
112	Endosulfan beta	4							85.36	23.81	19.45	115.99	3.80	3.28	88.35	9.64	10.91
113	Endosulfan sulfate	4							70.48	20.30	18.80	116.21	19.54	20.32	89.14	17.93	20.11
114	Endrin	4							88.96	17.68	22.54	107.57	12.66	11.77	92.69	12.73	13.73
115	Enrofloxacin	4							118.69	21.02	19.54	98.46	7.06	7.17	104.38	11.64	11.15
116	EPN	2				81.34	17.89	19.10	87.12	22.90	19.18	112.30	12.97	11.55	95.56	5.50	5.76
117	Epoxiconazole	0.8	122.98	16.59	13.49	89.44	11.43	13.96	97.21	19.34	17.51	106.20	12.49	11.76	106.31	5.68	5.34
118	Eprinomectin	1.2				100.03	21.36	21.35	89.96	19.07	21.20	95.57	10.18	10.65	102.43	8.65	8.44
119	Eritromicin	0.8	122.85	10.10	7.96	101.21	7.59	7.50	87.18	8.00	9.18	93.37	6.33	6.78	104.42	3.74	3.58
120	Esfenvalerate	2				127.54	21.58	16.92	105.91	19.49	17.83	101.51	4.20	4.14	90.24	7.45	8.26
121	Ethion (diethion)	0.8	109.21	7.32	6.70	92.19	7.99	8.67	89.52	8.27	9.24	98.50	2.63	2.67	100.66	3.50	3.48
122	Ethirimol	1.2				103.80	13.91	13.40	84.85	8.49	10.01	98.86	6.54	6.62	109.68	5.38	4.91
123	Ethofumesate	2				126.97	15.41	16.48	90.72	15.48	21.11	103.99	13.91	13.38	96.26	8.98	9.33
124	Ethoprophos	0.8	121.58	17.90	22.95	85.95	23.63	17.49	96.79	13.42	13.87	103.33	4.56	4.41	108.44	8.96	8.26
125	Etofenprox	1.2				103.05	20.35	19.75	88.00	12.88	14.64	87.54	4.70	5.37	107.43	8.42	7.84
126	Etoxazole	0.4	116.44	7.26	6.23	95.39	5.73	6.01	88.69	5.00	5.64	98.79	1.96	1.98	99.61	4.04	4.06
127	Famoxadone	1.2				105.50	21.91	19.80	84.93	8.09	9.53	100.06	8.50	8.49	105.60	8.62	8.16
128	Fenamidone	0.4	120.97	11.07	9.15	96.95	7.86	8.11	90.00	12.52	13.91	102.36	3.51	3.43	105.44	5.42	5.14
129	Fenamiphos	0.4	108.30	19.35	26.33	96.23	10.37	10.78	91.06	5.88	6.46	102.96	6.58	6.39	104.25	4.70	4.51
130	Fenamiphos sulfone	1.2				94.25	18.82	19.97	93.80	9.16	9.77	102.18	10.58	10.35	112.34	3.88	3.45
131	Fenamiphos sulfoxide	1.2				89.34	10.67	11.94	98.26	10.61	10.80	99.74	5.76	5.78	112.66	2.19	1.94
132	Fenarimol	1.6				100.34	12.81	12.77	99.76	7.71	7.73	109.44	6.21	5.67	94.11	1.40	1.49
133	Fenazaquin	0.4	120.20	17.57	14.62	91.49	3.55	3.88	82.11	9.35	11.39	100.13	2.89	2.89	101.08	3.40	3.36
134	Fenbendazole	0.4	120.77	22.32	17.07	98.07	9.36	9.54	84.88	12.32	14.51	99.37	8.88	8.94	105.25	5.15	4.89
135	Fenbuconazole	0.8	117.33	16.47	17.97	80.70	15.65	19.39	92.40	23.56	15.50	101.06	9.40	9.30	104.91	5.73	5.46
136	Fenhexamid	4							113.78	16.34	17.33	102.52	7.16	6.98	96.72	9.77	10.10
137	Fenitrothion	4							92.58	17.86	21.77	91.31	11.66	12.77	97.21	6.00	6.17
138	Fenoxycarb	0.4	105.61	17.40	16.48	94.00	12.18	12.96	87.03	6.88	7.91	101.05	3.54	3.50	99.09	6.54	6.60
139	Fenpropathrin	0.8	121.06	18.95	23.91	81.90	11.15	13.61	85.72	10.16	11.85	96.22	3.83	3.98	103.49	5.20	5.02
140	Fenpropidin	0.4	118.57	11.79	9.94	88.81	8.30	9.35	90.98	3.24	3.56	100.27	6.86	6.84	102.94	2.05	1.99
141	Fenpropimorph	0.8	118.79	13.41	11.29	85.42	10.61	12.42	89.45	8.55	9.56	101.39	8.15	8.04	103.06	4.18	4.06
142	Fenpyroximate	0.4	112.81	6.87	6.09	94.83	3.34	3.52	91.94	4.15	4.51	97.90	1.86	1.90	102.42	5.37	5.24
143	Fenthion	0.8	97.17	14.25	26.12	109.28	12.00	10.98	90.09	5.65	6.27	114.72	15.88	13.84	87.33	4.28	4.90
144	Fenthion oxon	0.4	112.27	10.39	9.25	93.67	4.79	5.11	87.28	8.70	9.97	102.19	5.74	5.62	105.28	3.24	3.08
145	Fenthion oxon sulfone	8										119.94	1.82	1.52	105.66	6.01	5.69
146	Fenthion oxon sulfoxide	1.2				104.05	10.41	10.00	88.97	11.85	13.32	101.42	5.20	5.13	107.05	4.90	4.58
147	Fenthion sulfone	1.6				111.17	8.93	8.03	95.30	19.79	20.77	105.00	10.11	9.63	105.57	6.32	5.99
148	Fenthion sulfoxide	1.6				115.59	18.20	15.75	93.36	12.69	13.59	104.11	6.72	6.45	105.76	5.54	5.24
149	Fenvalerate	2				107.17	18.70	19.85	88.71	2.41	2.72	133.74	8.81	6.59	89.16	9.12	10.23
150	Fipronil	0.8	102.09	11.72	11.48	94.23	19.32	19.56	93.24	8.74	9.37	97.93	6.41	6.55	101.30	2.10	2.07
151	Fipronil sulfide	8										99.25	16.95	17.08	101.31	10.48	10.34
152	Flocoumafen	0.4	106.82	17.50	16.38	94.93	7.63	8.04	84.00	8.55	10.18	101.02	3.52	3.48	102.48	3.21	3.13
153	Fluazinam	2				80.64	18.31	22.36	131.43	19.43	14.55	125.26	5.60	14.71	84.12	14.12	16.79
154	Flubendiamide	1.6				114.35	15.21	13.30	90.41	15.13	16.73	100.94	10.26	10.16	97.91	8.97	9.16
155	Flucythrinate (two isomers)	2				94.89	14.70	15.49	103.04	12.90	19.51	126.99	10.32	8.13	89.10	5.70	6.40
156	Fludioxonil	2				108.94	22.52	19.85	88.57	9.19	10.38	112.87	6.35	5.63	98.26	7.70	7.84
157	Flufenoxuron	0.8	118.74	20.77	17.49	90.49	6.24	6.90	83.45	5.81	6.96	100.22	5.17	5.16	102.52	5.23	5.10
158	Flumequine	0.8	127.04	7.72	5.63	91.69	7.33	7.99	90.22	9.12	10.11	95.00	6.52	6.86	104.99	4.54	4.32
159	Flunixin	0.8	123.19	8.48	6.88	99.80	18.40	18.44	86.25	12.61	14.62	93.81	5.62	5.99	103.21	8.75	8.48
160	Fluopyram	0.8	123.13	22.83	18.54	93.37	13.69	15.37	83.31	12.59	15.11	101.20	4.18	4.13	105.16	5.27	5.01
161	Fluoranthene	2				119.58	15.24	17.08	98.38	12.55	12.76	101.89	4.52	4.44	104.26	16.84	16.15
162	Fluorene	1.2				118.54	18.98	21.07	88.32	4.32	4.89	98.39	4.29	4.36	96.27	2.06	2.14
163	Fluquinconazole	1.2				110.55	16.66	18.74	86.38	6.85	7.93	100.01	12.08	12.08	81.40	7.90	9.71
164	Flusilazole	0.8	108.78	10.98	10.09	99.53	10.49	10.54	88.08	8.54	9.70	104.22	12.29	11.79	102.06	4.15	4.07
165	Flutolanil	0.8	117.13	16.40	14.00	99.03	10.07	10.17	90.65	12.51	13.80	100.14	3.12	3.12	102.55	7.51	7.32
166	Flutriafol	1.2				102.18	9.65	9.44	91.70	13.15	14.34	103.28	8.34	8.08	106.17	4.42	4.16
167	Fluvalinate tau	4							78.58	18.54	23.59	99.65	18.40	18.46	105.68	17.54	16.60
168	Fonofos	1.6				96.73	20.46	22.45	82.54	10.97	13.29	115.98	10.45	9.01	90.58	4.37	4.82
169	Formetanate	1.2				105.80	11.33	10.71	91.02	5.72	6.28	90.92	7.25	7.97	102.25	6.83	6.68
170	Fosthiazate	0.4	117.53	12.76	10.86	91.81	5.80	6.32	89.96	11.00	12.23	101.32	5.03	4.96	103.76	2.06	1.99
171	Heptachlor	1.2				105.63	13.94	13.20	95.68	11.66	12.19	109.43	7.97	7.28	91.44	3.65	3.99
172	Hexachlorobencene	0.8	98.45	17.43	18.64	107.10	20.87	20.35	87.60	4.82	5.50	101.47	9.94	9.80	90.60	2.38	2.63
173	Hexachlorocyclohexane (alpha)	2				117.54	12.12	8.21	88.98	5.41	6.08	103.02	9.89	9.60	92.16	8.25	8.95
174	Hexachlorocyclohexane (beta)	2				126.04	17.47	13.86	97.43	3.53	3.62	108.18	8.42	7.78	93.18	8.51	9.13
175	Hexachlorocyclohexane (delta)	4							90.15	19.06	20.45	104.74	16.22	15.49	95.22	11.94	12.54
176	Hexaclorocyclohexane (gamma, lindane)	4							93.75	14.15	23.24	107.48	6.80	6.33	95.82	12.95	13.51
177	Hexaconazole (two isomers)	1.6				95.19	15.08	15.84	91.80	7.62	8.30	102.65	5.70	5.55	103.19	2.94	2.85
178	Hexaflumuron	1.2				99.41	17.91	18.02	80.35	5.75	7.16	98.14	4.07	4.15	98.75	11.10	11.24
179	Hexythiazox	0.4	118.55	17.88	18.46	82.22	8.53	10.37	87.62	9.39	10.72	98.62	2.88	2.92	104.62	8.24	7.88
180	Imazalil (enilconazole)	0.8	130.92	15.49	17.18	95.75	7.74	8.08	89.57	9.74	10.87	100.82	4.01	3.98	104.51	5.19	4.97
181	Imidacloprid	4							117.55	5.49	4.67	107.18	8.37	7.81	102.44	8.24	8.04
182	Indeno [1,2,3-cd] pyrene	1.6				117.89	15.48	13.13	116.35	14.87	12.78	116.45	17.25	14.81	100.18	19.54	19.50
183	Indoxacarb	0.8	124.61	14.87	18.70	99.01	21.65	21.87	88.98	17.78	19.98	93.09	6.87	7.38	109.78	5.44	4.96
184	Iprovalicarb	0.8	115.20	10.97	9.52	93.14	7.55	8.11	89.11	6.56	7.36	103.80	5.49	5.29	102.29	3.92	3.83
185	Isofenphos methyl	2				126.11	13.75	10.90	96.55	9.27	9.60	112.12	5.55	4.95	96.12	9.68	10.07
186	Isoprothiolane	0.4	106.89	3.33	3.12	96.32	18.05	18.74	92.86	12.46	13.42	101.23	2.17	2.14	102.11	4.86	4.76
187	Ivermectin B1a	1.6				113.41	16.89	14.89	98.11	8.63	8.80	91.74	11.91	12.98	101.63	10.59	10.42
188	Josamycin	1.6				127.21	18.85	14.82	103.07	5.89	5.71	94.94	7.07	7.45	108.51	2.46	2.27
189	Ketoprofen	1.6				93.43	17.84	19.09	106.39	15.59	14.05	95.64	10.20	10.66	102.80	3.50	3.40
190	Kresoxim methyl	2				119.34	19.83	18.72	98.02	18.09	18.66	110.88	14.03	12.65	96.62	12.85	13.30
191	Levamisole	1.6				114.26	22.07	19.32	92.62	8.40	9.07	86.67	7.08	8.17	101.93	6.16	6.04
192	Lincomycin	4							120.69	19.64	16.27	112.23	7.04	6.27	96.55	6.39	6.62
193	Linuron	1.6				125.64	15.59	18.33	89.87	13.77	15.32	98.09	5.13	5.23	107.80	5.05	4.68
194	Lufenuron	0.8	112.88	16.42	14.55	97.32	13.82	14.48	78.06	11.50	14.73	102.81	10.91	10.61	102.87	4.15	4.03
195	Mandipropamid	0.4	110.19	14.68	13.32	91.62	7.61	8.31	91.88	8.21	8.94	103.64	4.69	4.53	104.43	3.29	3.15
196	Mebendazole	0.4	128.41	10.28	8.01	92.65	6.20	6.69	86.90	10.23	11.77	95.39	7.83	8.21	104.16	2.75	2.64
197	Mefenamic acid	1.6				123.97	17.95	14.48	102.51	18.77	18.31	92.91	13.78	14.83	102.92	3.83	3.72
198	Mefenoxam (metalaxyl-M)	0.4	119.22	10.17	8.53	93.63	6.26	6.69	91.63	10.08	11.00	101.70	6.18	6.08	103.38	2.08	2.01
199	Meloxicam	1.2				94.27	17.55	18.66	89.51	17.89	16.54	90.45	7.81	8.63	106.55	10.65	10.00
200	Mepanipyrim	2				114.71	20.02	17.45	92.36	6.47	7.01	105.72	8.41	7.95	102.76	2.90	2.82
201	Mepiquat	0.4	88.75	15.72	18.98	96.69	19.96	20.64	84.95	17.75	20.89	92.24	7.48	8.11	101.87	2.68	2.63
202	Metaflumizone	0.4	120.69	6.98	5.78	90.72	10.11	11.14	87.97	8.18	9.30	100.90	6.18	6.12	101.69	3.74	3.68
203	Metalaxyl	1.6				102.24	8.98	8.78	87.83	6.49	7.39	114.56	16.92	14.77	88.61	5.54	6.25
204	Metaldehyde	4							117.18	16.45	16.52	82.85	10.48	12.65	100.36	8.69	8.66
205	Metconazole	0.8	126.92	8.05	6.34	91.62	13.26	14.47	85.87	13.93	16.22	98.86	2.29	2.32	104.83	4.73	4.51
206	Methamidophos (two isomers)	8										90.08	12.33	13.69	95.62	7.09	7.41
207	Methidathion	0.4	121.63	11.90	9.78	94.81	6.86	7.24	92.10	11.60	12.60	101.70	2.15	2.11	105.46	7.13	6.76
208	Methiocarb	0.4	126.57	12.25	9.68	82.56	7.76	9.40	96.74	10.42	10.77	106.92	5.78	5.41	109.28	7.12	6.52
209	Methiocarb-sufone	2				124.44	19.05	20.07	110.41	9.03	8.18	106.55	7.79	7.31	105.17	9.43	8.97
210	Methiocarb-sulfoxide	1.2				97.90	11.90	12.16	97.45	19.13	19.63	102.57	5.80	5.65	107.53	5.47	5.09
211	Methomyl	1.2				105.76	12.28	11.61	116.29	18.70	16.08	114.17	3.66	3.21	106.03	7.48	7.05
212	Methoxyfenozide	0.4							86.63	21.88	23.34	102.29	15.53	15.18	97.40	9.54	9.79
213	Metoxychlor	4	116.96	8.59	7.34	88.75	7.33	8.26	90.85	3.70	4.07	102.21	3.91	3.83	102.30	3.36	3.28
214	Metrafenone	0.4	126.22	20.21	16.01	98.22	13.26	13.50	85.59	7.19	8.40	95.31	6.55	6.87	107.70	5.47	5.08
215	Metronidazole	12										77.85	19.32	22.63	94.46	8.54	9.04
216	Mevinphos (phosdrin)	1.2				107.14	19.43	18.14	87.21	9.70	11.12	99.51	6.93	6.96	112.13	4.49	4.00
217	Mirex	4							87.30	15.87	16.63	99.75	22.33	22.39	104.43	12.55	12.02
218	Monocrotophos	4							113.28	8.74	7.72	99.08	3.05	3.08	103.67	4.46	4.30
219	Moxidectin	4							97.05	17.46	19.81	97.15	19.05	19.61	101.68	7.42	7.30
220	Myclobutanil	0.8	104.83	19.53	19.31	99.69	13.23	13.27	87.82	10.56	12.02	102.16	9.43	9.23	104.53	3.52	3.37
221	N-(2.4-dimethylphenyl)-N′-methylformamidine (DMPF, metabolite of amitraz)	4							112.03	15.22	13.59	98.34	7.94	8.07	102.65	5.61	5.47
222	N.N-Dimethyl-N’-p-tolylsulphamide (DMST, metabolite of tolyfluanid)	4							115.30	9.04	7.84	106.19	4.77	4.49	106.16	3.58	3.37
223	Nafcillin	4							106.61	19.05	17.87	95.48	11.52	12.07	98.70	4.02	4.07
224	Naphtalene	1.6				82.87	15.67	17.47	114.46	17.98	14.45	106.51	18.61	17.47	96.49	6.52	6.76
225	Naproxen	2				128.28	18.41	16.21	112.22	2.15	18.76	113.75	19.60	17.23	102.62	6.86	6.68
226	Nitenpyram	8										109.62	19.55	17.83	100.70	5.77	5.73
227	Novobiocin	1.2				96.87	20.40	19.19	85.98	18.66	21.70	89.43	13.67	15.29	95.39	7.93	8.31
228	Nuarimol	1.2				106.98	17.30	15.52	92.46	7.78	8.41	122.94	10.78	8.77	89.25	5.47	6.13
229	Ofurace	0.8	118.87	20.45	6.62	93.50	18.17	19.43	88.50	9.60	10.85	94.99	4.06	4.27	110.23	4.44	4.03
230	Omethoate	2				106.78	14.85	13.91	97.86	17.25	17.63	87.65	6.69	7.63	105.07	13.17	12.53
231	Oxadixyl	0.8	123.61	18.91	15.30	97.19	4.29	4.41	85.77	7.12	8.30	98.57	5.69	5.77	106.26	2.50	2.35
232	Oxamyl	8										107.50	18.14	16.87	110.16	21.87	19.85
233	Oxamyl-oxime	8							99.19	10.92	11.01	80.81	9.57	11.84	93.86	10.65	11.35
234	Oxfendazole	0.8	132.57	17.25	13.01	100.86	3.40	3.37	90.01	13.08	14.53	95.72	9.30	9.72	105.18	2.02	1.92
235	Oxolinic acid	0.8							110.62	10.56	9.55	93.32	6.53	7.00	101.82	5.02	4.93
236	Oxydemeton methyl	4	125.26	11.57	8.95	103.93	6.92	6.66	90.21	15.13	16.77	94.20	6.15	6.53	103.58	4.77	4.61
237	Oxyfluorfen	4							87.69	18.03	21.96	104.71	11.43	10.92	97.01	14.18	14.62
238	Paclobutrazol	1.6				115.25	5.87	5.09	97.98	10.85	11.07	103.31	5.22	5.05	103.50	2.43	2.35
239	Parathion methyl	8										109.49	18.40	16.81	87.75	3.95	4.50
240	PCB 28	0.4	109.04	17.35	21.35	109.79	10.36	9.44	89.67	5.77	6.43	103.24	6.02	5.83	94.17	7.16	7.60
241	PCB 52	0.8	109.29	10.86	9.94	108.98	14.57	22.55	83.40	13.14	15.76	104.75	10.45	9.98	100.99	8.14	8.06
242	PCB 77	0.8	93.46	20.09	15.79	99.72	20.85	20.91	99.96	10.57	10.57	103.80	7.39	7.12	96.95	10.24	10.56
243	PCB 81	0.8	89.48	15.17	16.95	106.42	16.55	16.19	87.13	11.29	12.96	104.54	2.16	2.07	99.16	8.64	8.71
244	PCB 101	0.4	111.65	12.54	19.14	117.34	14.18	12.08	95.12	2.94	3.09	101.23	5.71	5.64	101.01	10.12	10.02
245	PCB 105	0.4	102.23	14.66	13.90	126.39	14.98	19.76	88.39	7.67	8.68	105.83	8.19	7.74	93.21	5.05	5.42
246	PCB 114	0.8	96.49	20.69	19.42	103.46	13.87	13.41	94.88	11.04	11.64	104.47	5.35	5.12	98.79	7.12	7.21
247	PCB 118	1.2				102.19	11.53	11.28	94.03	8.10	8.61	98.74	4.78	4.84	98.20	8.76	8.92
248	PCB 123	1.2				113.43	22.05	19.44	90.08	7.66	8.50	102.24	8.39	8.21	96.45	5.29	5.48
249	PCB 126	0.8	87.42	18.29	19.43	111.04	14.57	13.12	91.66	7.70	8.40	107.90	9.85	9.13	96.39	7.94	8.24
250	PCB 138	0.4	118.82	20.45	18.79	105.03	11.58	11.03	102.64	10.09	9.83	105.83	6.52	6.16	96.84	6.40	6.61
251	PCB 153	0.8	105.67	17.51	20.11	110.87	8.22	7.41	96.92	7.41	7.65	101.59	4.87	4.79	97.13	3.45	3.55
252	PCB 156	0.8	89.94	17.49	19.45	115.25	23.33	20.24	90.96	7.99	8.78	106.25	7.73	7.28	98.31	9.18	9.34
253	PCB 157	0.8	82.22	19.43	17.63	116.24	20.04	17.24	88.00	7.66	8.70	107.18	9.20	8.58	101.20	10.33	10.21
254	PCB 167	0.8	87.27	18.74	21.47	111.11	16.53	23.88	95.41	9.50	9.96	105.12	7.64	7.27	97.88	10.59	10.82
255	PCB 169	1.2				99.05	18.89	17.17	98.13	9.84	10.03	103.47	10.12	9.78	96.44	8.72	9.04
256	PCB 180	0.4	106.04	19.00	20.25	108.14	15.01	12.37	94.39	9.82	10.40	109.04	9.09	8.34	97.26	7.54	7.75
257	PCB 189	0.8	79.22	18.60	10.45	97.14	19.02	19.58	86.39	10.81	12.51	108.00	7.22	6.69	92.10	4.18	4.54
258	Penconazole	0.8	119.47	10.66	18.97	96.80	18.50	19.11	81.78	13.65	16.69	94.22	7.10	7.54	103.41	8.49	8.21
259	Pencycuron	0.4	110.82	13.26	11.97	90.36	11.63	12.87	86.17	10.68	12.39	98.98	3.84	3.88	101.16	6.82	6.74
260	Pendimethalin	2				102.88	19.86	19.30	75.06	16.21	21.60	104.56	5.38	5.15	94.40	11.99	12.70
261	Penicillin V	8										105.22	18.10	17.20	108.53	6.81	6.27
262	Permethrin	4							113.34	17.58	15.51	115.67	16.46	14.23	98.54	12.87	13.06
263	Phenanthrene	1.6				133.74	10.56	5.48	94.00	13.65	14.52	99.67	16.68	16.74	93.74	4.35	4.64
264	Phenylbutazone	16													110.32	17.68	21.84
265	Phosalone	0.4	110.50	17.38	15.73	98.28	8.36	8.51	89.91	5.27	5.86	101.98	2.80	2.75	102.70	2.35	2.29
266	Phosmet	1.2				100.60	6.02	5.98	92.49	12.42	13.43	101.79	5.92	5.82	104.63	5.42	5.18
267	Pthalamide (Folpet deg)	8										103.79	11.71	11.28	100.45	6.35	6.32
268	Pirimicarb	0.4	120.22	8.00	6.65	95.32	4.74	4.97	93.09	11.13	11.96	102.45	4.63	4.52	103.60	2.50	2.41
269	Pirimicarb-desmethyl	2				110.38	7.61	6.89	127.79	12.92	10.11	114.99	8.23	7.16	123.05	4.57	3.71
270	Pirimiphos ethyl	0.8	82.75	17.50	21.15	92.12	9.50	10.31	98.11	12.10	12.33	120.39	11.92	9.90	101.28	6.04	7.51
271	Pirimiphos methyl	0.4	111.79	15.98	14.29	95.45	16.02	16.78	88.67	10.14	11.44	102.62	4.30	4.19	101.90	6.74	6.61
272	Prochloraz	0.4	104.39	17.43	16.70	91.03	5.00	5.49	89.27	11.46	12.84	100.96	5.85	5.79	103.41	5.28	5.11
273	Procymidone	8										92.02	15.09	16.40	106.35	16.63	15.04
274	Profenofos	0.8	115.10	18.63	17.91	93.40	17.40	18.63	88.46	19.43	21.96	84.42	5.94	7.04	108.50	7.24	6.67
275	Propamocarb	2				114.94	19.15	16.66	93.17	2.45	2.63	99.37	9.65	9.71	103.21	7.96	7.71
276	Propargite	0.4	113.51	7.83	6.90	97.28	4.47	4.59	90.04	6.01	6.67	99.27	1.99	2.00	100.59	5.36	5.33
277	Propiconazole	2							103.21	16.19	15.38	98.49	10.53	10.69	103.63	3.30	3.18
278	Propoxur	0.8	124.75	20.23	15.08	92.34	8.00	8.66	93.26	18.90	20.27	101.62	8.07	7.94	111.03	3.68	3.31
279	Propyzamide (pronamide)	0.8	104.03	19.42	19.74	89.58	16.14	19.18	93.02	13.39	14.39	97.32	5.91	6.07	102.92	7.64	7.42
280	Proquinazid	1.6				102.99	15.84	15.38	92.93	3.72	4.00	116.31	13.68	11.76	95.04	8.78	9.24
281	Prothioconazol	1.6				118.94	17.99	15.13	86.94	10.77	12.39	101.86	7.03	6.90	103.49	5.02	4.85
282	Prothiophos	2				127.35	17.52	15.03	91.22	11.43	12.53	110.73	16.36	14.77	90.19	7.42	8.23
283	Pymetrozine	8										95.53	3.63	3.80	99.15	5.84	5.89
284	Pyraclostrobin	0.4	118.10	14.33	12.13	88.90	5.97	6.72	90.62	2.37	2.62	97.96	3.11	3.17	101.93	4.86	4.77
285	Pyrazophos	0.4	116.34	17.02	14.63	87.65	5.37	6.13	95.15	10.11	10.63	103.82	7.88	7.59	104.45	3.19	3.05
286	Pyrene	2				122.26	19.33	15.12	95.97	15.49	16.14	99.99	7.05	7.05	106.97	13.20	12.34
287	Pyridaben	0.4	111.80	8.50	7.60	96.96	11.34	11.70	89.29	7.37	8.25	97.16	3.54	3.64	101.94	5.96	5.85
288	Pyridaphenthion	0.4	125.33	10.87	8.67	88.86	12.18	13.71	90.69	9.17	10.11	103.36	5.75	5.56	104.97	2.29	2.18
289	Pyrimethanil	1.2				107.01	6.57	6.14	83.42	6.56	7.86	99.98	4.42	4.42	106.14	3.93	3.70
290	Pyriproxifen	0.4	108.80	6.84	6.29	93.68	5.81	6.20	84.68	4.33	5.11	99.26	4.88	4.92	97.21	10.36	10.66
291	Quinalfos	1.6				90.14	15.91	17.65	81.56	14.48	17.75	98.78	15.64	15.83	99.05	6.28	6.34
292	Quinoxyfen	0.8	106.79	20.65	20.64	102.29	14.68	14.27	92.06	12.98	14.10	113.55	3.75	3.30	88.93	8.36	9.40
293	Rifampicin	1.2				103.00	18.59	17.76	89.34	13.65	15.28	99.63	7.95	7.98	111.87	15.95	14.26
294	Rotenone	0.8	120.70	16.46	13.64	89.14	17.53	20.88	81.92	14.34	17.50	101.34	12.36	12.20	105.21	3.73	3.55
295	Roxithromycin	1.2				116.45	19.37	16.63	88.89	12.89	14.50	88.85	16.67	18.76	102.53	3.02	2.95
296	Sarafloxacin	20													102.28	16.90	16.52
297	Simazine	0.8	127.26	22.73	17.86	89.60	13.25	14.79	90.53	13.38	14.78	101.60	7.91	7.79	108.74	8.42	7.74
298	Spinosad (two isomers)	1.6	111.72	19.81	17.73	90.14	14.70	16.31	89.32	9.26	10.37	99.29	5.46	5.50	100.87	7.97	7.90
299	Spiramycin (two isomers)	12										105.21	2.31	2.20	98.92	6.55	6.62
300	Spirodiclofen	0.8	114.30	10.24	8.96	98.97	9.73	9.83	86.32	3.99	4.62	96.72	4.66	4.82	96.10	2.65	2.76
301	Spiromesifen	0.4	111.87	16.94	15.14	96.88	10.62	10.96	81.93	9.64	11.77	96.25	5.78	6.01	102.02	6.81	6.68
302	Spirotetramat	0.8	136.54	14.99	18.30	91.58	15.29	17.62	87.65	16.87	19.25	99.19	10.60	10.69	109.75	7.10	6.47
303	Spiroxamine	0.4	115.95	5.12	4.42	92.27	5.97	6.47	93.01	4.62	4.97	100.04	5.41	5.41	101.94	2.10	2.06
304	Strychnine	2				121.82	15.59	16.26	84.19	20.34	20.16	100.68	5.59	5.55	102.95	5.88	5.71
305	Sulfacetamide	16													94.80	9.96	10.51
306	Sulfachloropiridacine	4							109.37	18.24	16.68	98.92	7.84	7.93	102.01	6.91	6.77
307	Sulfadiacine	8										91.59	4.52	4.94	87.73	9.54	10.87
308	Sulfadimetoxine	2				126.54	15.11	10.31	109.89	11.08	10.08	103.69	8.01	7.72	103.97	3.74	3.60
309	Sulfadoxine	2				123.17	11.68	8.48	107.05	13.06	12.20	100.75	7.53	7.47	106.39	7.08	6.65
310	Sulfameracine	4							118.06	8.76	7.42	95.45	3.06	3.21	102.43	3.58	3.50
311	Sulfametacine	2				118.26	12.28	8.28	105.33	12.84	12.19	102.14	7.00	6.85	104.35	4.92	4.71
312	Sulfametizole	4							108.41	7.69	7.09	97.15	4.81	4.95	101.75	12.24	12.03
313	Sulfametoxazole	2				117.53	19.33	21.76	99.36	6.59	6.63	100.10	5.77	5.76	104.08	10.50	10.09
314	Sulfametoxipiridacine	1.6				122.56	10.02	7.23	93.76	15.04	16.04	95.82	4.12	4.30	106.04	6.02	5.68
315	Sulfamonomethoxine	4							115.82	19.10	16.49	109.03	9.00	8.25	102.71	5.89	5.73
316	Sulfapyridine	4							123.12	5.81	4.72	97.40	5.43	5.57	97.95	5.03	5.14
317	Sulfaquinoxaline	2				137.13	15.86	11.57	107.09	16.46	15.37	103.03	8.49	8.24	105.72	5.77	5.46
318	Sulfatiazole	4							114.09	12.17	10.67	89.17	3.00	3.36	96.75	9.66	9.98
319	Sulfisoxazole	4							123.17	15.55	12.62	109.15	6.85	6.28	102.56	9.83	9.58
320	Tebuconazole	2				124.33	10.86	8.73	102.25	9.82	9.60	103.18	10.27	9.95	100.50	6.55	6.52
321	Tebufenocide	0.8	113.40	19.30	17.02	94.01	6.42	6.83	88.38	6.07	6.87	99.16	2.48	2.50	99.92	5.26	5.26
322	Tebufenpyrad	0.8	125.31	18.01	14.37	106.17	5.54	5.22	88.47	11.68	13.20	96.73	4.95	5.12	106.06	2.21	2.08
323	Teflubenzuron	1.6				108.57	19.06	17.56	89.65	5.82	6.49	100.52	12.28	12.22	104.30	4.23	4.06
324	Tefluthrin	0.4	124.36	21.64	16.23	105.00	19.05	18.14	88.44	12.15	13.74	105.91	7.75	7.32	89.59	1.30	1.45
325	Telodrin (isobenzan)	2				121.92	12.85	21.31	88.03	14.49	17.82	108.27	5.89	5.44	99.09	17.36	17.52
326	Terbufos	0.8	104.09	14.21	13.26	88.68	15.21	16.32	81.58	8.12	9.95	110.15	9.52	8.64	95.99	5.50	5.73
327	Terbuthylazine	0.8	120.91	9.86	8.15	93.77	18.06	19.26	85.93	9.78	11.38	106.01	2.62	2.47	104.19	4.52	4.34
328	Tetrachlorvinphos	2				126.81	17.78	13.00	100.05	19.19	19.18	88.06	9.94	11.29	98.20	13.04	13.28
329	Tetraconazole	0.8	116.66	14.53	20.60	90.40	17.43	19.28	89.89	9.78	10.88	115.70	7.74	6.69	88.10	5.70	6.47
330	Tetradifon	1.6				106.92	12.99	19.50	91.97	11.87	12.91	112.56	5.80	5.15	94.92	2.68	2.82
331	Tetramethrin	2							120.30	14.89	12.38	113.61	8.45	7.44	99.35	15.78	15.88
332	Thiabendazole	1.2				96.72	17.21	16.26	86.00	6.75	7.85	109.00	4.57	4.19	95.59	7.21	7.54
333	Thiacloprid	0.8	121.34	7.44	5.66	90.02	6.38	7.09	88.41	14.87	16.82	108.68	3.58	3.29	110.31	5.93	5.38
334	Thiamethoxam	8										121.06	6.35	5.25	92.87	5.21	5.61
335	Thiophanate methyl	2				124.15	8.09	6.03	101.95	12.23	12.00	106.08	6.23	5.87	103.96	7.36	7.08
336	Tolclofos methyl	1.6				123.59	15.07	12.19	84.62	5.68	6.71	109.41	7.30	6.67	96.18	5.78	6.01
337	Tolfenamic acid	1.6				105.31	15.54	20.30	104.76	16.01	15.28	96.52	3.58	3.71	97.33	5.08	5.22
338	Triadimefon	1.2				94.15	15.09	16.33	95.62	7.48	7.82	101.84	6.27	6.16	103.91	2.78	2.68
339	Triadimenol	0.8	114.21	11.55	9.26	106.40	13.80	14.65	89.34	12.93	14.47	103.34	6.45	6.24	106.44	5.74	5.39
340	Triazophos (hostathion)	0.4	112.50	15.63	12.76	91.77	6.14	6.69	91.68	11.49	12.53	104.86	3.62	3.45	102.76	4.43	4.31
341	Trifloxystrobin	0.4	112.87	13.12	11.62	92.56	5.89	6.36	93.35	4.78	5.12	100.81	2.86	2.84	102.51	4.76	4.64
342	Triflumizole	0.4	110.08	9.26	8.41	94.01	7.01	7.46	93.26	7.62	8.17	99.91	2.69	2.69	101.62	3.67	3.61
343	Triflumuron	1.2				118.66	19.24	16.21	86.93	7.78	8.95	96.65	3.90	4.04	106.18	6.37	6.00
344	Trifluralin	1.2				93.29	17.78	19.06	92.40	6.37	6.89	105.89	9.83	9.28	93.54	4.17	4.46
345	Trimethoprim	2				116.02	16.54	12.16	109.21	15.05	13.78	94.20	12.51	13.28	97.92	8.70	8.88
346	Triticonazole	1.2				99.19	8.60	8.67	89.71	12.71	14.17	100.84	8.67	8.60	104.04	4.36	4.19
347	Tylmicosin	4							107.14	16.70	15.59	93.58	6.97	7.45	101.18	11.19	11.06
348	Tylosin	8										106.35	4.46	4.19	104.60	2.79	2.67
349	Vinclozolin	1.6				116.52	23.46	17.30	88.94	12.82	14.41	105.60	12.42	11.76	92.86	4.92	5.30
350	Warfarin	0.8	100.47	17.51	19.89	95.53	9.12	9.55	85.61	11.19	14.75	93.09	6.28	6.75	103.65	4.31	4.16
351	Zoxamide	0.8	121.96	19.52	18.47	88.50	11.13	13.88	90.57	19.33	20.34	106.93	5.18	4.84	105.40	6.44	6.11

## Appendix C

**Table A3:** Matrix Effect, expressed as percentage, calculated for all of the analytes. The range form −20% to 20% represent the tolerance range in which it is considered that no significant matrix effect exists.

No.	Compound	Matriz effect	No.	Compound	Matriz effect	Nº	Compound	Matriz Effect
1	2-Phenylphenol	−103.4	118	Eprinomectin	−6.3	235	Oxolinic acid	0.5
2	4,4′-Dichlorobenzophenone (metabolite of dicofol)	96.6	119	Eritromicin	45.4	236	Oxydemeton methyl	3.3
3	Abamectine	−70.6	120	Esfenvalerate	23.4	237	Oxyfluorfen	42.4
4	Acenaphthene	14.3	121	Ethion (diethion)	−13.7	238	Paclobutrazol	3.0
5	Acenaphtylene	17.0	122	Ethirimol	13.9	239	Parathion methyl	12.8
6	Acephate	−90.4	123	Ethofumesate	30.5	240	PCB 28	32.5
7	Acetamiprid	7.0	124	Ethoprophos	−20.5	241	PCB 52	32.7
8	Acrinathrin	16.2	125	Etofenprox	43.0	242	PCB 77	52.5
9	Albendazole	17.7	126	Etoxazole	−29.6	243	PCB 81	43.7
10	Aldicarb	5.8	127	Famoxadone	27.9	244	PCB 101	8.2
11	Aldicarb-sulfone	−4.5	128	Fenamidone	12.7	245	PCB 105	54.6
12	Aldicarb-sulfoxide	−2.9	129	Fenamiphos	14.0	246	PCB 114	46.6
13	Aldrin	43.7	130	Fenamiphos sulfone	10.4	247	PCB 118	45.0
14	Anthracene	23.9	131	Fenamiphos sulfoxide	0.0	248	PCB 123	45.7
15	Atrazine	7.8	132	Fenarimol	55.3	249	PCB 126	52.1
16	Azinphos-methyl	14.3	133	Fenazaquin	−108.8	250	PCB 138	54.9
17	Azoxystrobin	−6.9	134	Fenbendazole	−7.9	251	PCB 153	48.2
18	BDE-28	47.0	135	Fenbuconazole	67.7	252	PCB 156	59.3
19	BDE-47	47.8	136	Fenhexamid	5.9	253	PCB 157	51.6
20	BDE-85	43.7	137	Fenitrothion	54.6	254	PCB 167	60.1
21	BDE-99	57.3	138	Fenoxycarb	16.4	255	PCB 169	60.6
22	BDE-100	51.3	139	Fenpropathrin	−2.2	256	PCB 180	52.5
23	BDE-153	35.9	140	Fenpropidin	22.3	257	PCB 189	54.8
24	BDE-154	57.8	141	Fenpropimorph	1.7	258	Penconazole	50.4
25	BDE-183	−14.1	142	Fenpyroximate	19.8	259	Pencycuron	−37.4
26	Benalaxyl	−111.5	143	Fenthion	−33.9	260	Pendimethalin	29.8
27	Bendiocarb	−5.4	144	Fenthion oxon	27.1	261	Penicillin V	−8.1
28	Bendiocarb metabolite (2,2-dimethylbenzo-1,3-dioxol-4-ol)	39.7	145	Fenthion oxon sulfone	5.3	262	Permethrin	71.5
29	Benfuracarb	24.0	146	Fenthion oxon sulfoxide	3.7	263	Phenanthrene	29.6
30	Benzo[a]anthracene	58.5	147	Fenthion sulfone	6.9	264	Phenylbutazone	−4.9
31	Benzo[a]pyrene	45.0	148	Fenthion sulfoxide	11.9	265	Phosalone	−4.0
32	Benzo[b]fluoranthene	59.8	149	Fenvalerate	−39.6	266	Phosmet	5.8
33	Benzo[ghi]perylene	24.4	150	Fipronil	21.0	267	Pthalamide (Folpet deg)	59.3
34	Benzo[k]fluoranthene	38.1	151	Fipronil sulfide	676.5	268	Pirimicarb	1.7
35	Bifenthrin	80.4	152	Flocoumafen	−34.5	269	Pirimicarb-desmethyl	57.7
36	Bitertanol	−0.7	153	Fluazinam	−1.5	270	Pirimiphos ethyl	60.5
37	Boscalid (formerly nicobifen)	73.5	154	Flubendiamide	62.7	271	Pirimiphos methyl	−14.7
38	Brodifacoum	−31.7	155	Flucythrinate (two isomers)	4.0	272	Prochloraz	−37.2
39	Bromadiolone	−2.1	156	Fludioxonil	61.2	273	Procymidone	52.9
40	Bromopropylate	94.0	157	Flufenoxuron	1.1	274	Profenofos	−15.0
41	Bromuconazole (two isomers)	39.7	158	Flumequine	−0.8	275	Propamocarb	16.2
42	Bupirimate	57.0	159	Flunixin	−9.4	276	Propargite	−29.3
43	Buprofezin	−0.4	160	Fluopyram	45.4	277	Propiconazole	−85.8
44	Cadusafos (ebufos)	−54.8	161	Fluoranthene	36.0	278	Propoxur	−7.1
45	Carbaryl	15.8	162	Fluorene	17.2	279	Propyzamide (pronamide)	−2.0
46	Carbendazim (azole)	16.8	163	Fluquinconazole	55.3	280	Proquinazid	72.7
47	Carbofuran	−9.0	164	Flusilazole	3.8	281	Prothioconazol	51.5
48	Carbofuran-3-hydroxy	5.5	165	Flutolanil	−10.2	282	Prothiophos	49.5
49	Carbosulfan	−107.2	166	Flutriafol	54.5	283	Pymetrozine	16.2
50	Cefuroxima axetil (two isomers)	24.0	167	Fluvalinate tau	−31.7	284	Pyraclostrobin	−18.0
51	Chloramphenicol	24.4	168	Fonofos	28.9	285	Pyrazophos	20.6
52	Chlorantraniliprole	4.5	169	Formetanate	−38.7	286	Pyrene	45.1
53	Chlorfenvinphos	−31.3	170	Fosthiazate	2.9	287	Pyridaben	−83.6
54	Chlorobenzilate	95.2	171	Heptachlor	−110.9	288	Pyridaphenthion	14.3
55	Chlorophacinone	38.1	172	Hexachlorobencene	16.9	289	Pyrimethanil	30.6
56	Chlorpropham	24.2	173	Hexachlorocyclohexane (alpha)	−58.1	290	Pyriproxifen	−24.8
57	Chlorpyrifos	38.0	174	Hexachlorocyclohexane (beta)	−97.4	291	Quinalfos	3.7
58	Chlorpyrifos methyl	9.6	175	Hexachlorocyclohexane (delta)	−89.1	292	Quinoxyfen	−68.5
59	Chlorthal dimethyl	36.6	176	Hexaclorocyclohexane (gamma. lindane)	−109.8	293	Rifampicin	−68.9
60	Chrysene	38.1	177	Hexaconazole (two isomers)	−4.6	294	Rotenone	22.0
61	Clindamycin	−7.5	178	Hexaflumuron	−1.3	295	Roxithromycin	0.0
62	Clofentezine	13.2	179	Hexythiazox	−27.6	296	Sarafloxacin	114.8
63	Clothianidin	17.7	180	Imazalil (enilconazole)	11.5	297	Simazine	−19.7
64	Cloxacillin	4.9	181	Imidacloprid	4.1	298	Spinosad (two isomers)	−84.2
65	Coumachlor	46.2	182	Indeno [1,2,3-cd] pyrene	−132.0	299	Spiramycin (two isomers)	16.8
66	Coumaphos	−105.9	183	Indoxacarb	2.6	300	Spirodiclofen	−12.7
67	Coumatetralyl	−25.6	184	Iprovalicarb	−4.9	301	Spiromesifen	−23.6
68	Cyazofamid	12.7	185	Isofenphos methyl	42.0	302	Spirotetramat	−34.1
69	Cyflufenamid	−3.0	186	Isoprothiolane	−13.5	303	Spiroxamine	9.4
70	Cyfluthrin (sum of four isomers)	−6.1	187	Ivermectin B1a	−82.6	304	Strychnine	11.9
71	Cyhalothrin (lambda isomer)	−22.4	188	Josamycin	−0.4	305	Sulfacetamide	−27.9
72	Cymoxanil	13.6	189	Ketoprofen	10.0	306	Sulfachloropiridacine	61.1
73	Cypermethrin (sum of four isomers)	20.9	190	Kresoxim methyl	36.3	307	Sulfadiacine	−15.0
74	Cyproconazole (two isomers)	8.2	191	Levamisole	10.0	308	Sulfadimetoxine	18.2
75	Cyprodinil	−5.7	192	Lincomycin	135.3	309	Sulfadoxine	2.2
76	Cyromazine	−106.0	193	Linuron	0.0	310	Sulfameracine	83.7
77	Danofloxacin	172.1	194	Lufenuron	7.3	311	Sulfametacine	166.8
78	Dazomet	33.1	195	Mandipropamid	20.6	312	Sulfametizole	170.5
79	Deltamethrin	−33.7	196	Mebendazole	11.5	313	Sulfametoxazole	26.0
80	Demeton-S-methyl	−27.1	197	Mefenamic acid	33.5	314	Sulfametoxipiridacine	107.7
81	Demeton-S-methyl-sulfone (Dioxydemeton)	7.5	198	Mefenoxam (metalaxyl-M)	−5.9	315	Sulfamonomethoxine	1.7
82	Dexamethasone	5.4	199	Meloxicam	34.8	316	Sulfapyridine	24.6
83	Diazinon	22.3	200	Mepanipyrim	64.2	317	Sulfaquinoxaline	2.5
84	Dibenzo[a.h]anthracene	35.2	201	Mepiquat	−57.2	318	Sulfatiazole	32.5
85	Dichlorodiphenyldichloroethane (p,p′ DDD)	14.9	202	Metaflumizone	5.4	319	Sulfisoxazole	11.6
86	Dichlorodiphenyldichloroethylene (p,p′ DDE)	44.5	203	Metalaxyl	−47.0	320	Tebuconazole	−72.1
87	Dichlorodiphenyltrichloroethane (p,p′ DDT)	−124.9	204	Metaldehyde	−25.7	321	Tebufenocide	−7.8
88	Diclofenac	8.6	205	Metconazole	−26.1	322	Tebufenpyrad	7.9
89	Dicloran	−0.4	206	Methamidophos (two isomers)	−16.6	323	Teflubenzuron	−31.5
90	Diclorvos	−42.8	207	Methidathion	7.1	324	Tefluthrin	33.9
91	Dicloxacillin	−4.4	208	Methiocarb	6.2	325	Telodrin (isobenzan)	−22.0
92	Dieldrin	19.7	209	Methiocarb-sufone	3.0	326	Terbufos	24.0
93	Diethathyl ethyl	−15.6	210	Methiocarb-sulfoxide	3.0	327	Terbuthylazine	29.2
94	Diethofencarb	7.5	211	Methomyl	1.8	328	Tetrachlorvinphos	1.1
95	Difenacoum	7.8	212	Methoxyfenozide	−7.0	329	Tetraconazole	48.4
96	Difenoconazole	−21.3	213	Metoxychlor	−124.6	330	Tetradifon	41.7
97	Difethialone	16.8	214	Metrafenone	6.1	331	Tetramethrin	154.7
98	Difloxacin	99.9	215	Metronidazole	−13.3	332	Thiabendazole	67.7
99	Diflubenzuron	38.5	216	Mevinphos (phosdrin)	−3.3	333	Thiacloprid	12.3
100	Diflufenican	8.1	217	Mirex	−82.6	334	Thiamethoxam	19.7
101	Dimethenamid-P (and its R-isomer)	8.7	218	Monocrotophos	−1.2	335	Thiophanate methyl	−4.2
102	Dimethoate	4.8	219	Moxidectin	20.7	336	Tolclofos methyl	21.0
103	Dimethomorph (two isomers)	24.2	220	Myclobutanil	24.7	337	Tolfenamic acid	136.0
104	Dimethylphenylsulfamide (DMSA, metabolite of dichlofluanid)	4.9	221	N-(2,4-dimethylphenyl)-N′-methylformamidine (DMPF, metabolite of amitraz)	9.6	338	Triadimefon	20.6
105	Diniconazole-M	−11.4	222	N,N-Dimethyl-N′-p-tolylsulphamide (DMST, metabolite of tolyfluanid)	−8.2	339	Triadimenol	27.9
106	Dinocap	−11.9	223	Nafcillin	−8.2	340	Triazophos (hostathion)	−6.3
107	Diphacinone	14.1	224	Naphtalene	12.9	341	Trifloxystrobin	−55.8
108	Diphenylamine	−102.4	225	Naproxen	16.5	342	Triflumizole	−14.3
109	Dodine	−92.0	226	Nitenpyram	−4.5	343	Triflumuron	3.6
110	Doramectina	−16.2	227	Novobiocin	2.9	344	Trifluralin	−1.7
111	Endosulfan alfa	−2.5	228	Nuarimol	74.1	345	Trimethoprim	25.2
112	Endosulfan beta	−52.9	229	Ofurace	−27.2	346	Triticonazole	13.7
113	Endosulfan sulfate	−85.8	230	Omethoate	−0.4	347	Tylmicosin	140.3
114	Endrin	−59.9	231	Oxadixyl	6.5	348	Tylosin	−1.7
115	Enrofloxacin	141.8	232	Oxamyl	2.6	349	Vinclozolin	33.9
116	EPN	32.6	233	Oxamyl-oxime	30.1	350	Warfarin	0.0
117	Epoxiconazole	10.0	234	Oxfendazole	7.0	351	Zoxamide	−83.0
